# Genetic architecture and clinical features of Tourette syndrome in a child and adolescent cohort: an explorative clinical exome-based study

**DOI:** 10.3389/fpsyt.2026.1744145

**Published:** 2026-02-13

**Authors:** Federica Saia, Federica Mirabella, Andrea Longhitano, Nicoletta Maugeri, Rita Barone, Renata Rizzo

**Affiliations:** Child and Adolescent Neurology and Psychiatric Section, Azienda Ospedaliera Universitaria Policlinico ‘G. Rodolico-San Marco’, Department of Clinical and Experimental Medicine, University of Catania, Catania, Italy

**Keywords:** adolescents, children, neurodevelopmental disorders, Tourette syndrome, whole-exome sequencing

## Abstract

**Background:**

Tourette Syndrome (TS) is a neurodevelopmental disorder with a complex genetic architecture, involving both rare high-impact variants and polygenic contributions. While several risk genes have been identified by whole-exome sequencing (WES), the relationship between genetic variants and clinical phenotype in TS patients remains insufficiently characterized.

**Methods:**

We conducted an explorative clinical exome-based study in a cohort of 80 children and adolescents with TS (mean age 12.8 years; male:female = 70:10). Variants were classified as potentially causative (PC-Vs), variants of uncertain significance (VUS), or non-causative (NC-Vs) according to ACMG guidelines for variant evaluation and curated databases. Clinical assessment included tic severity through Yale Global Tic Severity Scale (YGTSS), cognitive testing, dysmorphic features, comorbid conditions, EEG and brain MRI analyses. Group comparisons were statistically performed to evaluate associations between genetic findings and phenotypic features.

**Results:**

Out of the 80 patients, 11 (13.7%) carried PC-Vs, 29 (36.3%) VUS, and 40 (50%) NC-Vs. Patients with PC-Vs exhibited significantly higher tic severity (mean YGTSS 28 ± 8.2) and lower IQ (67.6 ± 31.0) compared with VUS (YGTSS 24.2 ± 7.7; IQ 90.8 ± 23.1) and NC-Vs (YGTSS 18.5 ± 7.0; IQ 86.4 ± 20.3) (p < 0.05). Conversely, positive family history of tics was more frequent in the NC-V group (55%) than in PC-Vs (27.3%) or VUS (27.6%) (p = 0.044). Potential causative variants included *de novo* or inherited mutations in genes implicated in synaptic transmission (*PNKD*, *SLC6A1*), ion channels (*CACNA1D*), chromatin remodeling (*BRPF1*, *KMT2C*, *SMARCA2*), and pleiotropic neurodevelopmental pathways (*PTEN*, *RERE*).

**Conclusion:**

These findings support a dual model of genetic susceptibility in TS, where rare, high-impact exomic variants may contribute to more severe tics, cognitive impairment, and syndromic presentations, whereas polygenic inheritance mostly occurs in familial and milder forms. Incorporating exome sequencing into diagnostic workflows may enhance etiological classification and inform precision-medicine strategies for TS.

## 1 Introduction

Tourette Syndrome (TS) is a complex neurodevelopmental disorder characterized by persistent motor and vocal tics with an onset in childhood. It affects approximately 1% of the general population, with a male-to-female ratio of 3-4:1 (1–3).

The etiology of TS is multifactorial, involving a dynamic interplay between genetic, epigenetic, and environmental factors (4, 5). Twin and family studies support a strong heritable component and suggest that TS ranks among the neurodevelopmental disorders with the highest non-Mendelian transmission (6, 7).

Advances in next-generation sequencing, particularly whole-exome sequencing (WES), have substantially enhanced our understanding of the genetic architecture of TS, enabling the identification of both *de novo* and inherited rare coding variants that disrupt key neurodevelopmental pathways (8). These exomic variants include loss-of-function (LoF) and deleterious missense mutations in genes that are highly constrained and intolerant to variation (9).

Several WES studies in simplex trios have reported damaging *de novo* variants in approximately 10% of TS cases, with high-confidence risk genes such as *CELSR3*, *WWC1*, *NIPBL*, *FN1*, and *ASH1L* emerging as relevant contributors (8, 10). Beyond these core genes, recent research has highlighted the potential contribution of rare variants in candidate genes such as *PNKD* and *BRPF1*, which play key roles in presynaptic regulation and chromatin-mediated transcriptional control, respectively (11, 12). Variants in these genes may disrupt synaptic vesicle dynamics or epigenetic modulation, both of which are essential for maintaining the functional integrity of cortico-striatal-thalamo-cortical circuits implicated in tic generation. Moreover, WES meta-analyses have reported an enrichment of X-linked and autosomal rare variants, helping to explain the sex bias observed in TS and its frequent comorbidity with ASD and ADHD (8, 13).

Among these findings, a growing number of variants of uncertain significance (VUS), mutations for which the pathogenic potential remains unconfirmed, are being recognized as crucial to the expanding landscape of TS genetics (14). Despite their classification uncertainty, many VUS occur in genes with plausible neurobiological roles, such as those involved in ion channel activity (e.g., *CACNA1D*, *SLC6A1*) (15), GABAergic inhibition (16), chromatin remodeling, and neuronal migration (e.g., *RELN*, *ZMYM2*, *KMT2C*) (17–19). Importantly, some of these VUS may act as susceptibility loci or modifiers within a broader oligogenic or polygenic context, interacting with other rare or common variants to influence phenotypic expression and disease severity. Therefore, careful interpretation of VUS, especially those of *de novo* origin or affecting functionally constrained regions, is essential for refining the genotype–phenotype correlations in TS.

Building on this background, the present study aimed to clarify whether distinct genetic signatures can help stratify TS patients into clinically meaningful subtypes, with potential implications for early diagnosis and tailored interventions. In particular, we investigated whether potentially pathogenic exomic variants, including *de novo* variants and VUS, were associated with recurrent phenotypic features in a clinically well-characterized pediatric TS cohort.

By systematically analyzing the clinical and molecular profiles of affected individuals, we sought to identify genetic landmarks that may underlie distinct clinical subtypes of TS, contributing to the broader understanding of its heterogeneity and informing future precision medicine approaches.

## 2 Materials and methods

### 2.1 Participants

This study was conducted at the Child and Adolescent Neurology and Psychiatry of the Medical and Experimental Department, Catania University, Italy. Ancestry was assessed based on self-reported ethnicity, and all patients and their parents reported Italian ancestry. No genetic ancestry inference analyses (e.g., PCA) were performed. A total of 80 patients (average age 12.84, M: F 70:10), with a clinical diagnosis of TS according to DSM-5 criteria, have been enrolled for the study between April 2023 and April 2025.

We excluded patients with a primary psychiatric disorder different from TS, and/or presence of known etiology such as genetic or inherited metabolic diseases. Each participant was clinically assessed for tics and associated comorbidities. Investigations were carried out as part of the routine clinical care of the patients in accordance with the ethical standards laid down in the 1964 Declaration of Helsinki and its later amendments. Prior to enrolment, written informed consent was obtained from all participants’ parent or legal guardian. The study was approved by the local Ethics Committee (Catania 1) of Catania University Hospital (protocol code n° 67526, approved on December 3, 2024) for studies involving humans.

### 2.2 Procedures

Medical history was obtained from participant’s parents with a focus on developmental delay, seizures, neuropsychiatric disorders, and positive family history for neurodevelopmental disorder and metabolic diseases. All participants underwent physical and neurological examination. Fasting blood samples and urine samples were collected for routine blood analyses and to rule out inherited metabolic diseases. The occurrence of epileptic seizures and isolated electroencephalogram (EEG) anomalies were evaluated. Moreover, brain magnetic resonance imaging (MRI) using a 1.5 T MRI scanner, was examined to detect morphologically visible signs of altered brain development and atrophic alterations.

### 2.3 Clinical assessment

All participants underwent a full neuropsychiatric assessment for TS and related comorbidities. According to participants’ ages, the Wechsler Intelligence Scale for Children (WISC-IV) was administered to evaluate the intelligence quotient (IQ) of patients (20). Symptoms of TS were evaluated using the Yale Global Tic Severity Rating Scale (YGTSS) (21), a clinican-rated scale used to assess the motor and phonic tic severity considering the number, frequency, duration, intensity, and complexity of tics.

### 2.4 Genetic analysis

A clinical exome-based analysis was performed on DNA extracted from peripheral blood samples of the probands and the parents using the standard phenol extraction protocol. Library preparation for sequencing analysis was carried out using the SureSelect All Exon v6 kit (Agilent Technologies, Santa Clara, CA, USA), following the manufacturer’s instructions, with a focus on Neurodevelopmental Disorders (including both SNVs and CNVs).

Sequencing was conducted by using NGS technology with 100 bp paired-end reads and HiSeq 2500 platform (Illumina).

Bioinformatic analysis included sequence alignment to the human reference genome GRCh37/hg19 using the BWA algorithm (22), removal of non-specific alignments, variant calling through GATK (23), samtools, and bcftools, and quality control of target gene coverage and read depth within the requested gene panel (**Supplementary Table S1**). Although whole-exome sequencing (WES) technology was employed for data generation, variant interpretation followed a clinical exome workflow. Specifically, analysis was intentionally restricted to a curated virtual gene panel of genes associated with neurodevelopmental disorders, in accordance with routine clinical diagnostic practice, to prioritize clinically relevant findings and minimize false-positive and incidental results.

The clinical exome analysis covered 1,702 genes associated with neurodevelopmental disorders, corresponding to a total of 4,379,484 coding target bases. Mean depth of coverage (DoC) across target regions was 195.62x, while Max DoC was 5,832.22x, with an average of 99.29% ± 0.41 of target bases covered at ≥20×, 99.72% ± 0.13 at ≥10× and 99.87% ± 0.06 at ≥5x. Given the limited inter-sample variability, sequencing quality metrics were summarized descriptively as mean ± standard deviation across the cohort.

Variant filtering was performed using the following criteria: minimum read depth ≥10×, genotype quality ≥20, and allele balance ≥0.25 for heterozygous variants. Variants with a minor allele frequency (MAF) >1% in population databases (gnomAD, 24) were excluded from further analyses. Selected variants were confirmed by Sanger sequencing. Variant interpretation was conducted according to the guidelines from the American College of Medical Genetics and Genomics (ACMG) as a general framework for variant evaluation (25), while final variant prioritization and categorization were performed using an integrative approach described below. Identified variants were annotated using population databases such as GnomAD v.4.1.0, Exome Aggregation Consortium (ExAC) (26), 1000 genome database (27), Exome Variant Server (EVS), and clinical genetic databases, i.e. OMIM (28), ClinVar (29), HGMD (30), GWAS (31). Each variant was subjected to in silico analysis to predict its impact on protein structure and/or function, as well as its evolutionary conservation, using tools such as PolyPhen-2 (32), SIFT (33), CADD (34), MutationTaster (35), MutationAssessor (36), and PhyloP (37).

### 2.5 Exome data analysis and variants classification

Variant prioritization and classification were performed using a multi-step approach integrating both variant-level and gene-level evidence. At the variant level, the following parameters were considered: (i) variant type (e.g. missense, nonsense, frameshift, splice-site, and *de novo* variants); (ii) minor allele frequency (MAF) in population databases; (iii) in silico pathogenicity prediction scores (including CADD, SIFT, PolyPhen-2, and REVEL, when available); and (iv) predicted impact on protein function and evolutionary conservation.

Subsequently, variants were evaluated at the gene level based on known gene–disease associations, with particular attention to genes previously implicated in Tourette syndrome or other neurodevelopmental and neuropsychiatric disorders. Final classification into “Potentially Causative” variants (PC-V), “Variants of Uncertain Significance” (VUS), and “Non-causative Variants” (NC-V) was achieved by integrating variant-level evidence with gene-level information, and was further refined through consultation of the scientific literature and public and private genomic databases, including the database of genomic variants (http://dgv.tcag.ca/dgv/app/home), the Decipher database (https://decipher.sanger.ac.uk/), the database of human genomic structural variation (https://www.ncbi.nlm.nih.gov/dbvar), and the OMIM catalogue (http://www.ncbi.nlm.nih.gov/omim).

In the present study, variants classified as “Potentially Causative” (PC-V) were those showing a combination of low population frequency, high predicted pathogenic impact, and a plausible biological relevance, and occurring in genes previously associated with Tourette syndrome or related neurodevelopmental and neuropsychiatric disorders, as supported by OMIM and the scientific literature.

Variants were classified as “Variants of Uncertain Significance” when they fulfilled variant-level criteria suggestive of potential functional relevance but lacked sufficient or consistent evidence for a definitive association with Tourette syndrome, being only sporadically reported in the literature or occurring in genes broadly implicated in neuropsychiatric phenotypes.

“Non-causative” variants (NC-V) were defined as variants with no evidence of association with Tourette syndrome in the literature or databases, and/or showing limited predicted functional impact. Whole-exome sequencing was performed using a trio-based approach, including the proband and both biological parents, allowing the assessment of variant inheritance and the identification of *de novo* variants. Whenever PC-V or VUS variants were identified, parental segregation analysis was used to support variant interpretation and genetic counselling.

In some cases, extended segregation analyses beyond the proband–parent trio could not be completed due to parental unavailability, adoption, or mono-parental family structure.

### 2.6 Statistical analysis

Children with PC-V, VUS, and NC-V were statistically compared to verify the effect of presence/absence of variants on dysmorphic features, comorbidities, ID, YGTSS scores, as well as on the rate of epilepsy, EEG anomalies and brain MRI anomalies. Clinical characteristics of participants were summarized by randomized group using mean (SD) for continuous data or count and percentage (%) for categorical data. Pairwise comparisons were performed using one-way ANOVA or the Kruskal–Wallis test for continuous variables, according to data distribution, and the chi-square test (χ²) for categorical variables. The Kolmogorov-Smirnov test was applied to assess data distribution and guide the choice between parametric and non-parametric methods. A p-value < 0.05 was considered to indicate statistical significance. When the χ² test or ANOVA/Kruskal-Wallis test showed significant results, *post hoc* pairwise comparisons were conducted, with a Bonferroni correction applied to control for potential inflation of Type I error. After this adjustment, the significance threshold (alpha) was set at 0.017. Data were analyzed using GraphPad Prism version 10.0.0 (GraphPad Software, San Diego, CA, USA).

## 3 Results

### 3.1 Genetic findings

Among the 80 children with TS, 11 patients (8 males and 3 females) were classified as having PC-Vs (13.75%), 29 patients (25 males and 4 females) as having VUS (36.25%), and 40 patients (37 males and 3 females) as carrying NC-Vs (50%) (**Table 1**).

**Table 1:** Demographic and genetic characteristics of TS patients.

Patient group	Patients (*n*)	Age (Years) (mean ± SD)	M: F ratio	Percentage/all
All	80	12.84 ± 4.65	70:10	100%
Patients with PC-Vs	11	14.45 ± 5.32	8:3	13.75%
Patients with VUS	29	12.03 ± 4	25:4	36.25%
Patients with NC-Vs	40	12.98 ± 4.88	37:3	50%

TS, Tourette Syndrome; PC-Vs, potentially causative variants; VUS, variants of uncertain significance; NC-Vs, non-causative variants; F, female; M, male; *n*, number; SD, standard deviation.

Eleven variants were classified as pathogenic/causative variants, six of which were identified as *de novo*. The clinical and genetic features associated with PC-Vs, including variant type, minor allele frequency (MAF), and pathogenicity prediction scores (CADD), are summarized in **Table 2**. Corresponding analyses for Variants of Uncertain Significance and Non-causative Variants are reported in **Supplementary Tables S2**, **S3**, respectively. Variant classification was based on an integrative framework combining variant-level characteristics (variant type, population frequency, and pathogenicity prediction scores, when available) and gene-level evidence, as detailed in the Methods section. Sequencing quality metrics demonstrated high and homogeneous coverage across the cohort, ensuring reliable detection of rare coding variants.

**Table 2:** Clinical and molecular characteristics associated with PC-Vs in patients with Tourette syndrome.

Patient ID	Main gene(s) involved	Type of variant	MAF	CADD score	Clinical features
#1	*KMT2C*	*De novo* frameshift, c.10400dup p.(Pro3468ThrfsTer27)	N/A	N/A	Low IQ (72), dysmorphic features, ADHD
#2	*PNKD*	Heterozygous deletion, c.76_103del p.(Ala26IlefsTer38)	0.00004	N/A	MRI anomalies, EEG anomalies, OCD, anxiety
#3	*BRPF1*	*De novo* missense, c.1545C>G p.(Asp515Glu)	N/A	22.9	ADHD
#4	*PNKD*	heterozygous missense, c.1126C>T	0.00006	32	No major findings
#5	*SMARCA2*	*De novo* missense, c.1279C>T p.(Arg427Cys)	N/A	32	Dysmorphic features, specific learning disorder
#6	*CACNA1D* *RERE*	Heterozygous missense, c.3439G>A p.(Glu1147Lys) in *CACNA1D* gene; Heterozygous in-frame deletion, c.3925_3954del p.(Ile1309_Glu1318del) in *RERE* gene	N/A	27.3	Developmental delay, epilepsy, ADHD, Intellectual disability, dysmorphic features, behavioral problems
#7	*MAPK8IP3*	*De novo* missense, c.3299G>T p.(Arg1100Leu)	0.000004	30	Language disorder, motor tics, learning difficulties
#8	*SLC20A2*	Heterozygous missense, c.541C>T p.(Arg181Trp)	~0.0000	18.58	MRI anomalies, dysmorphic features, OCD
#9	*PTEN*	*De novo* nonsense, c.1003C>T p.(Arg335Ter)	N/A	48	Low IQ (66), MRI anomalies, dysmorphic features, ADHD
#10	*ANKRD17*	*De novo* missense variant, c.6961C>T p.(Arg2321Cys)	0.00001	27.3	Low IQ (75), dysmorphic features, motor/vocal tics within a broader neurodevelopmental syndrome
#11	*SLC6A1*	*De novo* missense, c.1195C>G p.(Cys399Arg)	N/A	N/A	Epilepsy, anxiety, OCD, ADHD

MAF, minor allele frequency; CADD score, Combined Annotation Dependent Depletion prediction score; ADHD, attention-deficit/hyperactivity disorder; OCD, obsessive–compulsive disorder; MRI, magnetic resonance imaging; EEG, electroencephalography; IQ, intelligence quotient; N/A, not available due to absence of the variant in population databases or lack of annotation by prediction tools.

Not all variants were associated with complete population frequency or in silico prediction data due to variant type and database coverage; missing annotations (N/A) reflect intrinsic limitations of population and prediction databases for rare genetic variation.

### 3.2 Genetic mapping statistics per individual exome

For each proband, clinically reported variants retained after quality control and clinical interpretation were extracted from finalized clinical exome reports (**Supplementary Table 4**).

Across the cohort, the number of reported variants per individual ranged from 1 to 5, including both single-nucleotide variants (SNVs) and small insertions/deletions (indels). The majority of reported variants were coding variants, with a variable proportion of rare variants (MAF <1%) across individuals.

These clinically curated variants represent the subset used for genotype–phenotype correlation analyses and were classified according to variant type, rarity, and predicted functional impact.

### 3.3 Clinical characteristics of the cohort

In this study, we recruited 80 patients (mean age = 12.84 ± 4.65; male/female ratio = 70/10) with a clinical diagnosis of TS, as determined by the Yale Global Tic Severity Rating Scale (YGTSS).

**Figure 1:**
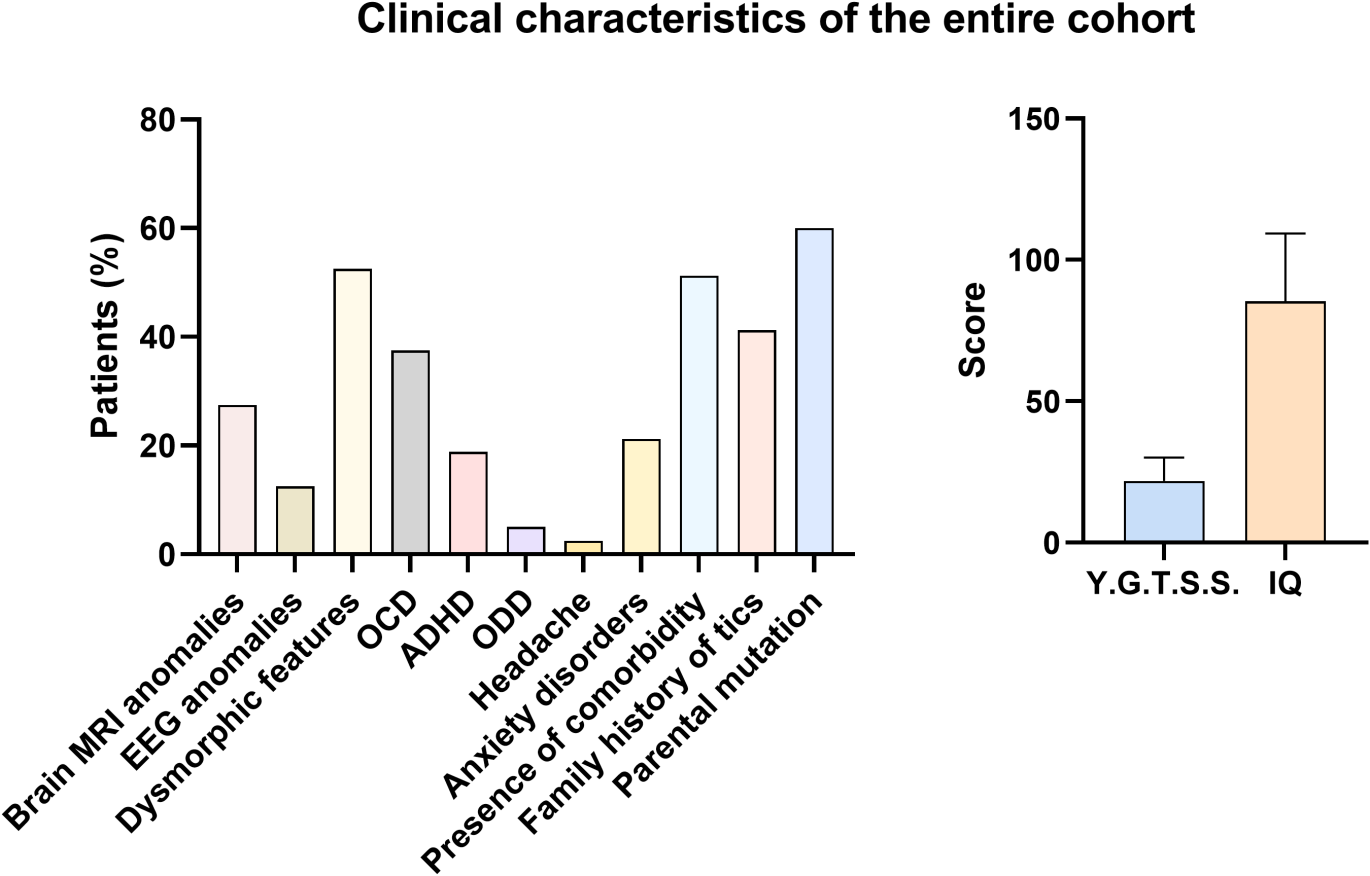
Frequency of clinical features of the Tourette syndrome cohort. Continuous variables are reported as mean ± standard deviation (SD), whereas categorical variables are expressed as percentage (%).

The overall clinical and neuropsychiatric characteristics of the cohort are summarized in **Table 3**, while the frequency distribution of the main phenotypic features is illustrated in **Figure 1**.

**Table 3:** Clinical and neuropsychiatric characteristics of the entire cohort of patients with Tourette syndrome.

Clinical feature	Total cohort (80)
Y.G.T.S.S.	21.84 ± 8.16
IQ	85.29 ± 24.04
EEG anomalies	10 (12.5%)
Dysmorphic features	42 (52.5%)
Brain MRI anomalies	22 (27.5%)
Obsessive–compulsive disorder (OCD)	30 (37.5%)
Attention-deficit/hyperactivity disorder (ADHD)	15 (18.8%)
Oppositional defiant disorder (ODD)	4 (5%)
Headache	2 (2.5%)
Anxiety disorders	17 (21.2%)
Presence of comorbidity	41 (51.2%)
Family history of tics	33 (41.2%)
Parental mutation	48 (60%)

TS, Tourette syndrome; Y.G.T.S.S., Yale Global Tic Severity Scale; IQ, intelligence quotient; EEG, electroencephalography; MRI, magnetic resonance imaging; OCD, obsessive–compulsive disorder; ADHD, attention-deficit/hyperactivity disorder; ODD, oppositional defiant disorder.

Continuous variables are reported as mean ± standard deviation (SD), while categorical variables are expressed as number and percentage (n, %).

The mean total YGTSS score was 21.84 ± 8.16, indicating an overall moderate tic severity. Cognitive functioning was heterogeneous, with a mean IQ of 85.29 ± 24.04, reflecting a substantial proportion of patients with borderline or impaired intellectual functioning.

Among the observed clinical features, dysmorphic traits (52.5%) and the presence of at least one psychiatric comorbidity (51.2%) were the most frequently detected characteristics in the cohort. Obsessive–compulsive disorder represented the most common psychiatric comorbidity (37.5%), followed by anxiety disorders (21.2%) and attention-deficit/hyperactivity disorder (18.8%), confirming the high burden of internalizing and neurodevelopmental symptoms in pediatric Tourette syndrome. The prevalence of a positive family history of tics (41.2%) further underscores the relevance of inherited factors in a substantial proportion of cases.

### 3.4 Clinical characteristics of the cohort stratified by variant classification

**Figure 2:**
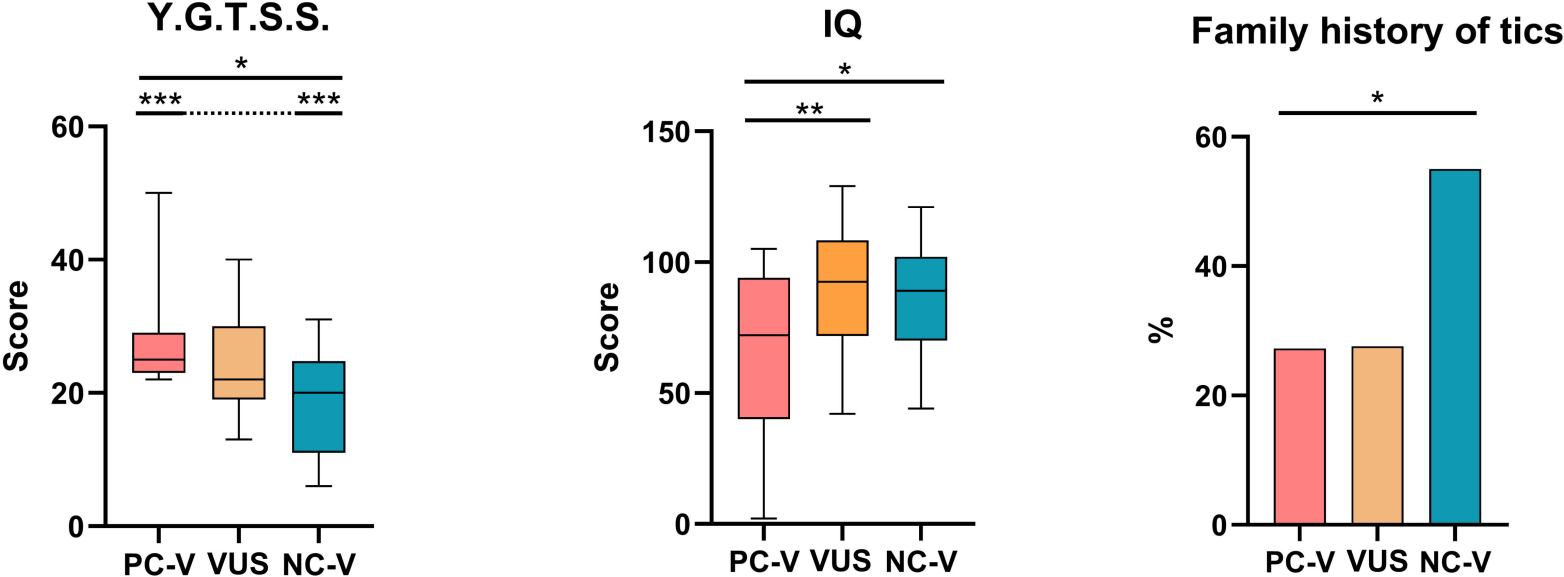
Clinical variables with statistically significant differences among TS children, according to variant classification. Paired *post hoc* comparisons with Bonferroni adjustment were used. *p < 0.05; **p < 0.017; ***p < 0.008. PC-Vs, potentially causative variants; VUS variants of uncertain significance; NC-Vs, non-causative variants.

**Table 4** reports the results of χ² tests and ANOVA/Kruskal–Wallis analyses comparing clinical and neuropsychiatric features among patients classified as carrying potentially causative variants (PC-Vs), variants of uncertain significance (VUS), or non-causative variants (NC-Vs). Significant pairwise differences are illustrated in **Figure 2**.

**Table 4:** Somatic and neuropsychiatric features in patients with TS according to variant classification.

Clinical feature	PC-Vs (11)	VUS (29)	NC-Vs (40)	*p-value*
Y.G.T.S.S.	28 ± 8.21	24.17 ± 7.7	18.45 ± 6.98	**0.001^c^**
Brain MRI anomalies	3 (27.27%)	10 (34.5%)	9 (22.5%)	0.546^a^
EEG anomalies	1 (9.09%)	6 (20.7%)	3 (7.5%)	0.245^a^
Dysmorphic features	6 (54.55%)	18 (62.1%)	18 (45%)	0.371^a^
IQ	67.55 ± 31.02	90.79 ± 23.08	86.36 ± 20.25	**0.020** ^b^
OCD	2 (18.18%)	11 (37.9%)	17 (42.5%)	0.336^a^
ADHD	3 (27.27%)	6 (20.7%)	6 (15%)	0.617^a^
ODD	0	2 (6.9%)	2 (5%)	0.671^a^
Headache	0	0	2 (5%)	0.359^a^
Anxiety	2 (18.18)	7 (24.1%)	8 (20%)	0.885^a^
Presence of comorbidities	3 (27.27%)	17 (58.6%)	21 (52.5%)	0.203^a^
Family history of tics	3 (27.27%)	8 (27.6%)	22 (55%)	**0.044** ^a^
Parental mutations	5 (45.45%)	21 (72.4%)	22 (55%)	0.197^a^

TS, Tourette syndrome; PC-Vs, potentially causative variants; VUS, variants of uncertain significance; NC-Vs, non-causative variants; Y.G.T.S.S., Yale Global Tic Severity Scale; IQ, intelligence quotient; OCD, obsessive–compulsive disorder; ADHD, attention-deficit/hyperactivity disorder; ODD, oppositional defiant disorder; EEG, electroencephalography; MRI, magnetic resonance imaging.

Superscript “a” indicates results obtained with the Chi-square (χ²) test; superscript “b” indicates results obtained with one-way analysis of variance (ANOVA); superscript “c” indicates results obtained with Kruskal–Wallis test.

Data are presented as means ± standard deviations or as counts and percentages. Statistically significant values (p < 0.05) are indicated in bold.

#### 3.4.1 Tic severity

The severity of tic symptoms, assessed with the Yale Global Tic Severity Scale (YGTSS), showed a progressive gradient: children with potentially causative variants (PC-Vs) exhibited the highest mean scores (28 ± 8.21), followed by those with variants of uncertain significance (VUS, 24.17 ± 7.7), and finally the non-causative group (NC-Vs), which displayed the lowest scores (18.45 ± 6.98). The intergroup difference reached statistical significance (p = 0.001), indicating that the presence of potentially pathogenic variants is associated with greater clinical burden in terms of tic manifestation.

#### 3.4.2 Cognitive evaluation

Mean IQ was markedly reduced in the PC-V group (67.55 ± 31.02), compared with both VUS (90.79 ± 23.08) and NC-Vs (86.36 ± 20.25). The differences between groups were statistically significant (p = 0.020), suggesting that rare high-impact variants may contribute not only to tic severity but also to broader neurodevelopmental impairment.

#### 3.4.3 EEG anomalies

Electroencephalographic abnormalities were documented in a minority of patients, with frequencies ranging from 7.5% in NC-Vs to 20.7% in VUS and 9.1% in PC-Vs. Despite these variations, the overall comparison did not reach statistical significance (p = 0.245), suggesting that EEG alterations are not systematically linked to variant type in this cohort.

#### 3.4.4 MRI analysis

Structural brain anomalies identified on MRI were relatively common but comparably distributed across groups. They were present in 27.3% of PC-V patients, 34.5% of VUS, and 22.5% of NC-Vs, without significant group differences (p = 0.546). These findings suggest that, while MRI alterations are not uncommon in TS, they do not appear to segregate according to the genetic classification adopted in this study.

#### 3.4.5 Dysmorphic features

Dysmorphic features were observed with notable frequency across all subgroups, ranging from 45% in NC-Vs to 62.1% in VUS carriers. However, these variations did not reach statistical significance (p = 0.371). This indicates that, although dysmorphisms are frequent in pediatric TS patients, they cannot be specifically attributed to one genetic subgroup.

#### 3.4.6 Comorbidities

Psychiatric comorbidities were highly prevalent in the cohort, though their distribution did not significantly differ among genetic subgroups. Obsessive–compulsive disorder (OCD) was most frequently reported in the NC-V group (42.5%), followed by VUS (37.9%), and less commonly in PC-Vs (18.2%) (p = 0.336). Attention-deficit/hyperactivity disorder (ADHD) showed the opposite trend, being most frequent among PC-V carriers (27.3%), compared with 20.7% in VUS and 15% in NC-Vs (p = 0.617). Other comorbidities such as oppositional defiant disorder (ODD), anxiety, and headache occurred at lower rates overall, without significant intergroup differences. The overall prevalence of psychiatric comorbidity was highest in the VUS (58.6%) and NC-Vs (52.5%) subgroups, compared to 27.3% in the PC-V group (p = 0.203).

#### 3.4.7 Family history of tics

A statistically significant difference emerged when analyzing the distribution of family history of tics across the three subgroups (p = 0.044). In the PC-V and VUS groups, familial aggregation was relatively uncommon, being reported in only 27.3% and 27.6% of patients, respectively. In contrast, more than half of the NC-V subgroup (55%) had at least one family member with a history of tic disorders.

## 4 Discussion

By integrating genetic findings with detailed neuropsychiatric assessments, this study aimed to provide novel insights into the contribution of rare exomic variants, including both *de novo* and inherited mutations, to the clinical heterogeneity of Tourette syndrome.

Our cohort of 80 children and adolescents with TS was characterized by a predominance of males (male-to-female ratio 7:1) and a mean age of 12.8 years, in line with epidemiological data showing the higher prevalence and earlier onset of tics in boys (2, 3).

From a neurobiological and genetic perspective, sex differences in TS and related neurodevelopmental disorders have been interpreted within the framework of sex-specific liability thresholds and the female protective effect. According to this model, females may require a higher cumulative genetic burden to manifest clinically overt disease, a mechanism supported across multiple neurodevelopmental conditions and increasingly suggested by exome-based studies in tic-related phenotypes (8).

Across the sample, tics ranged from mild to severe, and psychiatric comorbidities such as obsessive–compulsive disorder (OCD), attention-deficit/hyperactivity disorder (ADHD), anxiety, and oppositional defiant disorder (ODD) were frequent, reflecting the well-established clinical spectrum of TS (38). Dysmorphic traits, intellectual disability, and epilepsy were also observed in a relevant proportion of patients, especially those carrying potentially causative variants (PC-Vs). MRI anomalies were present in about a quarter of the cohort, and EEG abnormalities in around 10–20%, findings consistent with the variable but nonspecific neurological alterations described in the literature (39).

Genetic subgrouping revealed clear clinical distinctions. Children with PC-Vs displayed the highest tic severity scores on the Yale Global Tic Severity Scale (YGTSS), along with significantly lower IQ values compared to those with VUS or non-causative variants. In contrast, patients without candidate variants more often reported a positive family history of tics, supporting the hypothesis that inherited polygenic burden drives familial clustering, while *de novo* high-effect variants underlie more severe simplex and syndromic presentations. These demographic and clinical patterns reinforce the dual model of TS genetic architecture, in which rare variants act as strong risk factors for severe phenotypes, and common variants contribute to broader familial risk (8, 40, 41). Importantly, the classification of variants into potentially causative, uncertain, or non-causative categories was not based solely on gene–disease associations but relied on an integrative framework combining variant-level features (including variant type, population frequency, and in silico pathogenicity predictions) with gene-level evidence. This approach was adopted to reduce misclassification and to account for the heterogeneous nature of rare genetic variation in neurodevelopmental disorders.

Building on these observations, the detailed analysis of individual PC-V cases provided further insight into how specific genes contribute to tic severity, cognitive outcome, and associated comorbidities.

Patient 1, carrying a *de novo* truncating variant in *KMT2C*, presented with borderline intellectual functioning, dysmorphic features, ADHD, and Tourette syndrome. Pathogenic *KMT2C* variants have been associated with developmental delay and dysmorphism (42, 43), but tics have not been systematically described, suggesting that our case expands the known phenotype. Patient 2, harboring a truncating variant in *PNKD*, showed severe tics with OCD and anxiety but preserved cognition, a pattern strikingly similar to the familial cases reported by Sun et al. (11).

By contrast, Patient 4, with a missense variant in the same gene, displayed only moderate tics without comorbidities, exemplifying the uncertainty in interpreting non-truncating variants in synaptic genes.

Variants in chromatin regulators also emerged as relevant contributors. Patient 3, with a *de novo BRPF1* variant, presented with ADHD and tics but preserved cognition and no dysmorphism, diverging from the more syndromic presentations described in the literature (12). Patient 5 carried a *SMARCA2* missense variant, typically linked to Nicolaides–Baraitser syndrome with severe intellectual disability and seizures (44), but in our case associated with preserved IQ, a learning disorder, and moderate tics. These cases illustrate how chromatin-remodeling genes, while generally linked to syndromic neurodevelopmental disorders, may predispose to tic phenotypes in milder allelic presentations.

Some patients presented complex phenotypes involving multiple or pleiotropic gene effects. Patient 6 carried dual variants in *CACNA1D* and *RERE* and exhibited developmental delay, epilepsy, and tics. Both genes are individually implicated in neurodevelopmental disorders (45, 46), and their co-occurrence suggests additive or oligogenic effects, as previously hypothesized in TS cohorts with multiple rare variants (40).

Similarly, Patient 7 carried a *de novo* variant in *MAPK8IP3*, a gene associated with intellectual disability and axonal transport dysfunction (47). Although tics have not been reported in *MAPK8IP3*-related disorders, their presence in our case suggests that axonal transport defects may also predispose to movement disorders, extending the clinical spectrum of this condition.

Other findings highlighted interpretative challenges. Patient 8 harbored a heterozygous missense variant in *SLC20A2*, classically linked to adult-onset primary familial brain calcification (48). In this child, the absence of calcifications renders the variant more consistent with a VUS, analogous to CNV findings in pediatric TS where variants in late-onset genes are difficult to classify. In contrast, Patient 9 carried a *de novo PTEN* nonsense mutation and exhibited macrocephaly, dysmorphism, intellectual disability, ADHD, and tics, a constellation highly consistent with PTEN hamartoma tumor syndrome (49). Patient 10, with a *de novo ANKRD17* variant, showed dysmorphism, borderline cognition, and tics. While *ANKRD17* has been linked to intellectual disability and behavioral disturbances (50), tics have not been reported, suggesting an expansion of the phenotypic spectrum.

Finally, Patient 11 carried a *de novo* missense variant in *SLC6A1* and presented with epilepsy, ADHD, OCD, anxiety, and tics. Pathogenic *SLC6A1* variants are well-recognized causes of epilepsy with developmental delay and psychiatric comorbidities (51). The strong phenotypic overlap with published cases supports impaired GABAergic inhibition as a convergent mechanism in Tourette syndrome (40).

Taken together, these cases highlight recurring themes: truncating and *de novo* variants in constrained genes are associated with more severe and syndromic phenotypes, while inherited or missense variants remain more challenging to interpret but may still act as modifiers. The overlap with genes implicated in broader neurodevelopmental syndromes underscores the pleiotropy and shared vulnerability across psychiatric and neurodevelopmental disorders. Psychiatric comorbidities such as OCD, ADHD, and anxiety were frequent in all groups but did not significantly differ by variant type, suggesting they are more strongly influenced by polygenic and environmental factors (38). Likewise, MRI and EEG anomalies did not differentiate between genetic subgroups, consistent with clinical guidelines that do not recommend routine imaging in typical TS (39).

Our findings underscore the complementary contributions of rare high-effect variants and inherited polygenic burden in the pathogenesis of Tourette syndrome. Patients with potentially pathogenic variants tend to exhibit higher tic severity, lower cognitive scores, and syndromic features, while familial cases without such variants reflect polygenic inheritance. Comprehensive genomic testing is particularly warranted in patients with atypical or syndromic features, while future studies integrating rare and common variant analyses with longitudinal phenotyping and functional validation will be critical to refine variant interpretation, improve classification frameworks, and develop mechanism-driven interventions.

## 5 Conclusions

This study explored the genetic basis and clinical heterogeneity of Tourette Syndrome in a pediatric cohort by integrating clinical exome sequencing with detailed phenotypic evaluation. The findings confirm the dual contribution of genetic mechanisms: on one side, rare, high-impact variants were associated with more severe clinical outcomes, including higher tic severity, lower cognitive scores, and syndromic features; on the other, the absence of clearly pathogenic variants correlated more strongly with familial aggregation, suggesting a polygenic or inherited background.

From a molecular perspective, the study highlighted the involvement of diverse neurodevelopmental pathways, including chromatin remodeling, synaptic transmission and inhibitory signaling, ion channel function and pleiotropic regulators. These results reinforce the overlap between TS and other neurodevelopmental disorders and support the notion of a shared genetic vulnerability across conditions.

Despite these insights, several limitations should be acknowledged. The relatively small sample size limits the statistical power and generalizability of the findings. Moreover, the classification of variants, particularly variants of uncertain significance, remains challenging and may evolve as new functional and population data become available. The lack of systematic parental testing in all cases also restricted the interpretation of inheritance patterns in some families.

From a clinical standpoint, the study underscores the diagnostic utility of exome sequencing in complex or atypical cases of TS. Identifying rare, potentially causative variants may help guide patient management, genetic counseling, and risk stratification, especially in children presenting with intellectual disability, dysmorphic traits, epilepsy, or structural brain anomalies. At the same time, recognition of polygenic inheritance in familial cases emphasizes the importance of considering both rare and common variants in clinical practice.

## Data Availability

The raw data supporting the conclusions of this article will be made available by the authors, without undue reservation.
