# Supplementary material for: Genetic architecture and clinical features of Tourette syndrome in a child and adolescent cohort: an explorative clinical exome-based study
Source: Front Psychiatry. 2026 Feb 13;17:1744145. doi: 10.3389/fpsyt.2026.1744145 (PMC12946004; doi:10.3389/fpsyt.2026.1744145)
Supplement: Supplementary file 1 [file Table1.docx]

Supplementary Material

**Supplementary Table 1.** Virtual gene panel of 1,702 genes associated with neurodevelopmental disorders used for clinical exome–based analysis.

| *AARS1* | *CNPY3* | *GMNN* | *MRAS* | *PTRH2* | *TET3* |
| --- | --- | --- | --- | --- | --- |
| *AARS2* | *CNTNAP1* | *GMPPA* | *MRE11* | *PTRHD1* | *TFE3* |
| *ABCA2* | *CNTNAP2* | *GMPPB* | *MRM2* | *PUF60* | *TGIF1* |
| *ABCC9* | *COA8* | *GNAI1* | *MRPS22* | *PUM1* | *TH* |
| *ABCD1* | *COASY* | *GNAO1* | *MRPS34* | *PURA* | *THG1L* |
| *ABCD4* | *COG1* | *GNAS* | *MRTFB* | *PUS1* | *THOC2* |
| *ABCE1* | *COG5* | *GNB1* | *MSL3* | *PUS3* | *THOC6* |
| *ABHD16A* | *COG6* | *GNB2* | *MSMO1* | *PUS7* | *THRA* |
| *ABHD5* | *COG7* | *GNB5* | *MSTO1* | *PYCR1* | *THRB* |
| *ACAD9* | *COG8* | *GNPAT* | *MTFMT* | *PYCR2* | *THUMPD1* |
| *ACADM* | *COL18A1* | *GNPTAB* | *MTO1* | *QARS1* | *TIAM1* |
| *ACADS* | *COL27A1* | *GNPTG* | *MTOR* | *QRICH1* | *TIMM50* |
| *ACBD6* | *COL3A1* | *GNS* | *MTR* | *RAB11A* | *TIMM8A* |
| *ACO2* | *COL4A1* | *GOLGA2* | *MTRFR* | *RAB11B* | *TIMMDC1* |
| *ACOX1* | *COL4A2* | *GOT2* | *MTRR* | *RAB18* | *TKFC* |
| *ACSF3* | *COLEC11* | *GPAA1* | *MVK* | *RAB23* | *TKT* |
| *ACSL4* | *COLGALT1* | *GPC3* | *MYCBP2* | *RAB27A* | *TLE3* |
| *ACTB* | *COPB1* | *GPC4* | *MYCN* | *RAB39B* | *TLK2* |
| *ACTG1* | *COPB2* | *GPHN* | *MYH10* | *RAB3GAP1* | *TMCO1* |
| *ACTL6A* | *COQ2* | *GPT2* | *MYO5A* | *RAB3GAP2* | *TMEM106B* |
| *ACTL6B* | *COQ4* | *GRIA1* | *MYRF* | *RABGAP1* | *TMEM107* |
| *ACVR1* | *COQ8A* | *GRIA2* | *MYT1L* | *RAC1* | *TMEM147* |
| *ACY1* | *COX10* | *GRIA3* | *NAA10* | *RAC3* | *TMEM163* |
| *ADAM22* | *COX15* | *GRIA4* | *NAA15* | *RAD21* | *TMEM165* |
| *ADAR* | *CPE* | *GRID2* | *NAA20* | *RAF1* | *TMEM216* |
| *ADARB1* | *CPLANE1* | *GRIK2* | *NACC1* | *RAI1* | *TMEM218* |
| *ADAT3* | *CPLX1* | *GRIN1* | *NAE1* | *RALA* | *TMEM222* |
| *ADD1* | *CPSF3* | *GRIN2A* | *NAGA* | *RALGAPA1* | *TMEM231* |
| *ADD3* | *CPSF7* | *GRIN2B* | *NAGLU* | *RAP1B* | *TMEM237* |
| *ADGRG1* | *CRADD* | *GRIN2D* | *NALCN* | *RAP1GDS1* | *TMEM240* |
| *ADGRL1* | *CRBN* | *GRM1* | *NANS* | *RARB* | *TMEM63A* |
| *ADK* | *CREBBP* | *GRM7* | *NAPB* | *RARS1* | *TMEM63B* |
| *ADNP* | *CRIPT* | *GSS* | *NARS1* | *RARS2* | *TMEM63C* |
| *ADPRS* | *CRLS1* | *GSX2* | *NARS2* | *RBBP8* | *TMEM67* |
| *ADSL* | *CRMP1* | *GTF2E2* | *NAXD* | *RBL2* | *TMEM70* |
| *AFF2* | *CRPPA* | *GTF2H5* | *NAXE* | *RBM10* | *TMEM94* |
| *AFF4* | *CSDE1* | *GTPBP2* | *NBEA* | *RBM28* | *TMTC3* |
| *AGA* | *CSF1R* | *GTPBP3* | *NBN* | *RBPJ* | *TMX2* |
| *AGAP1* | *CSGALNACT1* | *GUSB* | *NCAPG2* | *RCBTB1* | *TNIK* |
| *AGMO* | *CSNK1G1* | *H1-4* | *NCDN* | *RELN* | *TNPO2* |
| *AGO1* | *CSNK2A1* | *H3-3A* | *NCKAP1* | *RERE* | *TNR* |
| *AGO2* | *CSNK2B* | *H3-3B* | *NCOR1* | *RFT1* | *TNRC6B* |
| *AGPAT2* | *CSPP1* | *H4C3* | *NDE1* | *RFX3* | *TOE1* |
| *AGPS* | *CTBP1* | *H4C5* | *NDP* | *RFX4* | *TOGARAM1* |
| *AGTPBP1* | *CTCF* | *H4C9* | *NDST1* | *RFX7* | *TOP3A* |
| *AHCY* | *CTDP1* | *HACE1* | *NDUFA1* | *RGS6* | *TOPORS* |
| *AHDC1* | *CTNNA2* | *HARS1* | *NDUFA11* | *RHOBTB2* | *TOR1A* |
| *AHI1* | *CTNNB1* | *HAX1* | *NDUFA2* | *RIC1* | *TOR1AIP1* |
| *AIFM1* | *CTNND1* | *HCCS* | *NDUFA6* | *RIMS1* | *TP53RK* |
| *AIMP1* | *CTR9* | *HCFC1* | *NDUFAF2* | *RIMS2* | *TP73* |
| *AIMP2* | *CTU2* | *HCN1* | *NDUFAF3* | *RIT1* | *TPI1* |
| *AK1* | *CUL3* | *HCRT* | *NDUFAF4* | *RLIM* | *TPK1* |
| *AKT3* | *CUL4B* | *HDAC4* | *NDUFAF5* | *RMND1* | *TPP2* |
| *ALDH18A1* | *CUX1* | *HDAC8* | *NDUFAF6* | *RNASEH2A* | *TPRKB* |
| *ALDH3A2* | *CUX2* | *HEATR3* | *NDUFAF8* | *RNASEH2B* | *TRA2B* |
| *ALDH5A1* | *CWC27* | *HECTD4* | *NDUFB3* | *RNASEH2C* | *TRAF7* |
| *ALDH7A1* | *CWF19L1* | *HECW2* | *NDUFB8* | *RNASET2* | *TRAIP* |
| *ALG1* | *CYB5R3* | *HEPACAM* | *NDUFC2* | *RNF113A* | *TRAK1* |
| *ALG11* | *CYC1* | *HERC1* | *NDUFS1* | *RNF125* | *TRAPPC10* |
| *ALG12* | *CYFIP2* | *HERC2* | *NDUFS2* | *RNF13* | *TRAPPC11* |
| *ALG13* | *CYP26B1* | *HESX1* | *NDUFS3* | *RNF220* | *TRAPPC12* |
| *ALG2* | *CYP27A1* | *HGSNAT* | *NDUFS4* | *RNFT2* | *TRAPPC2L* |
| *ALG3* | *CYP2U1* | *HIBCH* | *NDUFS7* | *RNH1* | *TRAPPC4* |
| *ALG6* | *D2HGDH* | *HID1* | *NDUFS8* | *RNPC3* | *TRAPPC6B* |
| *ALG9* | *DAG1* | *HIKESHI* | *NDUFV1* | *ROBO1* | *TRAPPC9* |
| *ALKBH8* | *DAGLA* | *HIRA* | *NDUFV2* | *ROGDI* | *TREX1* |
| *ALS2* | *DARS1* | *HIVEP2* | *NEB* | *ROR2* | *TRIM8* |
| *AMER1* | *DARS2* | *HK1* | *NECAP1* | *RORA* | *TRIO* |
| *AMFR* | *DBT* | *HLCS* | *NECTIN1* | *RPGRIP1L* | *TRIP12* |
| *AMPD2* | *DCAF17* | *HMGB1* | *NEDD4L* | *RPL10* | *TRIT1* |
| *AMT* | *DCC* | *HNMT* | *NEMF* | *RPS6KA3* | *TRMT1* |
| *ANAPC1* | *DCHS1* | *HNRNPD* | *NEU1* | *RRAGC* | *TRMT10A* |
| *ANK2* | *DCPS* | *HNRNPH1* | *NEURL4* | *RRAS2* | *TRMT5* |
| *ANK3* | *DCX* | *HNRNPH2* | *NEUROD2* | *RRM2B* | *TRNT1* |
| *ANKRD11* | *DDB1* | *HNRNPK* | *NEUROG1* | *RSPRY1* | *TRPC5* |
| *ANKRD17* | *DDC* | *HNRNPR* | *NEXMIF* | *RSRC1* | *TRPM3* |
| *ANKS1B* | *DDHD2* | *HNRNPU* | *NF1* | *RTEL1* | *TSC1* |
| *ANO10* | *DDOST* | *HNRNPUL2* | *NFASC* | *RTN4IP1* | *TSC2* |
| *ANO3* | *DDX23* | *HOXA1* | *NFIA* | *RTTN* | *TSEN15* |
| *ANP32A* | *DDX3X* | *HPCA* | *NFIB* | *RUNX1T1* | *TSEN2* |
| *AP1B1* | *DDX59* | *HPD* | *NFIX* | *RUSC2* | *TSEN54* |
| *AP1G1* | *DDX6* | *HPDL* | *NFU1* | *SALL1* | *TSFM* |
| *AP1S1* | *DEAF1* | *HPRT1* | *NGLY1* | *SAMD9* | *TSPAN7* |
| *AP1S2* | *DEGS1* | *HRAS* | *NHLRC2* | *SAMHD1* | *TSPOAP1* |
| *AP2M1* | *DENND5A* | *HS2ST1* | *NHS* | *SART3* | *TTC5* |
| *AP3B1* | *DEPDC5* | *HSD17B10* | *NIPBL* | *SASS6* | *TTC8* |
| *AP3B2* | *DHCR24* | *HSD17B4* | *NKAP* | *SATB1* | *TTI1* |
| *AP3D1* | *DHCR7* | *HSPD1* | *NKX2-1* | *SATB2* | *TTI2* |
| *AP4B1* | *DHDDS* | *HTRA2* | *NKX6-2* | *SBDS* | *TUBA1A* |
| *AP4E1* | *DHFR* | *HTT* | *NLGN3* | *SC5D* | *TUBB* |
| *AP4M1* | *DHPS* | *HUWE1* | *NLGN4X* | *SCAF4* | *TUBB2A* |
| *AP4S1* | *DHTKD1* | *IARS1* | *NLRP3* | *SCAMP5* | *TUBB2B* |
| *APC2* | *DHX30* | *IDH2* | *NMNAT1* | *SCAPER* | *TUBB3* |
| *APTX* | *DHX37* | *IDS* | *NONO* | *SCN10A* | *TUBB4A* |
| *ARCN1* | *DHX9* | *IDUA* | *NOVA2* | *SCN1A* | *TUBG1* |
| *ARF1* | *DIAPH1* | *IER3IP1* | *NPHP1* | *SCN1B* | *TUBGCP2* |
| *ARF3* | *DIS3L2* | *IFIH1* | *NPRL2* | *SCN2A* | *TUBGCP6* |
| *ARFGEF1* | *DKC1* | *IFT172* | *NR2F1* | *SCN3A* | *TUSC3* |
| *ARFGEF2* | *DLAT* | *IFT74* | *NR4A2* | *SCN8A* | *TWIST1* |
| *ARG1* | *DLD* | *IGF1* | *NRAS* | *SCO2* | *TWIST2* |
| *ARHGAP32* | *DLG3* | *IGF1R* | *NRCAM* | *SCYL1* | *TWNK* |
| *ARHGAP35* | *DLG4* | *IKBKG* | *NRROS* | *SCYL2* | *U2AF2* |
| *ARHGEF9* | *DLL1* | *IL1RAPL1* | *NRXN1* | *SDCCAG8* | *UBA2* |
| *ARID1A* | *DMD* | *IMPA1* | *NSD1* | *SDHA* | *UBA5* |
| *ARID1B* | *DMPK* | *IMPDH2* | *NSD2* | *SDHAF1* | *UBAP2L* |
| *ARID2* | *DMXL2* | *INPP4A* | *NSDHL* | *SDHB* | *UBE2A* |
| *ARL13B* | *DNAH14* | *INPP5E* | *NSRP1* | *SDHD* | *UBE3A* |
| *ARL3* | *DNAJC12* | *INPP5K* | *NSUN2* | *SEMA6B* | *UBE3B* |
| *ARL6* | *DNAJC19* | *INTS1* | *NSUN6* | *SEPSECS* | *UBE4A* |
| *ARMC9* | *DNASE2* | *INTS11* | *NT5C2* | *SERAC1* | *UBR1* |
| *ARPC4* | *DNM1* | *IPO8* | *NTNG2* | *SET* | *UBR7* |
| *ARSB* | *DNM1L* | *IPP* | *NTRK1* | *SETBP1* | *UBTF* |
| *ARSL* | *DNMT3A* | *IQSEC1* | *NTRK2* | *SETD1A* | *UFC1* |
| *ARV1* | *DNMT3B* | *IQSEC2* | *NUBPL* | *SETD1B* | *UFM1* |
| *ARX* | *DOCK3* | *IRF2BPL* | *NUDT2* | *SETD2* | *UFSP2* |
| *ASH1L* | *DOCK6* | *IRX5* | *NUP107* | *SETD5* | *UGDH* |
| *ASL* | *DOCK7* | *ISCA2* | *NUP188* | *SFXN4* | *UGP2* |
| *ASNS* | *DOCK8* | *ITCH* | *NUP214* | *SGPL1* | *UNC45A* |
| *ASPA* | *DOHH* | *ITGAV* | *NUS1* | *SGSH* | *UNC79* |
| *ASPM* | *DONSON* | *ITPA* | *NUSAP1* | *SHANK2* | *UNC80* |
| *ASXL1* | *DPAGT1* | *ITPR1* | *OCLN* | *SHANK3* | *UPF3B* |
| *ASXL2* | *DPF2* | *ITSN1* | *OCRL* | *SHH* | *UQCC2* |
| *ASXL3* | *DPH1* | *IVD* | *ODC1* | *SHMT2* | *USP27X* |
| *ATAD3A* | *DPH5* | *JAM2* | *OFD1* | *SHOC2* | *USP7* |
| *ATG7* | *DPM1* | *JAM3* | *OGDH* | *SHQ1* | *USP9X* |
| *ATIC* | *DPM3* | *JARID2* | *OGDHL* | *SHROOM4* | *VAC14* |
| *ATN1* | *DPP6* | *KANSL1* | *OGT* | *SIAH1* | *VAMP2* |
| *ATP13A2* | *DPYD* | *KARS1* | *OPHN1* | *SIK1* | *VARS1* |
| *ATP1A1* | *DPYS* | *KAT6A* | *ORC1* | *SIL1* | *VARS2* |
| *ATP1A2* | *DPYSL5* | *KAT6B* | *OSGEP* | *SIN3A* | *VLDLR* |
| *ATP1A3* | *DRG1* | *KAT8* | *OTUD5* | *SIN3B* | *VPS11* |
| *ATP2A2* | *DSCAM* | *KATNB1* | *OTUD6B* | *SIX3* | *VPS13B* |
| *ATP2B1* | *DTYMK* | *KATNIP* | *OTX2* | *SKI* | *VPS13D* |
| *ATP5F1A* | *DYM* | *KCNA1* | *OXR1* | *SKIC3* | *VPS16* |
| *ATP5F1E* | *DYNC1H1* | *KCNA2* | *P4HTM* | *SLC12A2* | *VPS35L* |
| *ATP5PO* | *DYNC1I2* | *KCNA6* | *PABPC1* | *SLC12A5* | *VPS37A* |
| *ATP6AP2* | *DYRK1A* | *KCNB1* | *PACS1* | *SLC12A6* | *VPS41* |
| *ATP6V0A1* | *EARS2* | *KCNC1* | *PACS2* | *SLC13A5* | *VPS4A* |
| *ATP6V0A2* | *EBF3* | *KCNC2* | *PAFAH1B1* | *SLC16A2* | *VPS53* |
| *ATP6V0C* | *EBP* | *KCND2* | *PAH* | *SLC17A5* | *VRK1* |
| *ATP6V1A* | *ECHS1* | *KCNH1* | *PAK1* | *SLC18A2* | *WAC* |
| *ATP6V1B2* | *EDEM3* | *KCNH5* | *PAK3* | *SLC1A2* | *WARS1* |
| *ATP7A* | *EED* | *KCNJ10* | *PALS1* | *SLC1A4* | *WARS2* |
| *ATP8A2* | *EEF1A2* | *KCNJ11* | *PAN2* | *SLC25A1* | *WASF1* |
| *ATP9A* | *EEF1B2* | *KCNJ6* | *PANK2* | *SLC25A12* | *WASHC4* |
| *ATR* | *EEF1D* | *KCNK4* | *PARN* | *SLC25A15* | *WBP4* |
| *ATRX* | *EEF2* | *KCNK9* | *PARP6* | *SLC25A19* | *WDFY3* |
| *AUH* | *EFL1* | *KCNMA1* | *PARS2* | *SLC25A22* | *WDR11* |
| *AUTS2* | *EFTUD2* | *KCNN2* | *PAX5* | *SLC25A42* | *WDR26* |
| *B3GALNT2* | *EHMT1* | *KCNN3* | *PAX6* | *SLC2A1* | *WDR37* |
| *B3GALT6* | *EIF2AK2* | *KCNQ2* | *PBX1* | *SLC30A7* | *WDR4* |
| *B3GAT3* | *EIF2AK3* | *KCNQ3* | *PC* | *SLC30A9* | *WDR45* |
| *B3GLCT* | *EIF2B1* | *KCNQ5* | *PCDH12* | *SLC32A1* | *WDR45B* |
| *B4GALNT1* | *EIF2B2* | *KCNT1* | *PCDH19* | *SLC33A1* | *WDR5* |
| *B4GALT1* | *EIF2B3* | *KCNT2* | *PCDHGC4* | *SLC35A2* | *WDR62* |
| *B4GALT7* | *EIF2B4* | *KCTD7* | *PCGF2* | *SLC35A3* | *WDR73* |
| *B9D1* | *EIF2B5* | *KDM1A* | *PCLO* | *SLC35B2* | *WDR81* |
| *BAP1* | *EIF2S3* | *KDM2B* | *PCNT* | *SLC35C1* | *WIPI2* |
| *BBS1* | *EIF3F* | *KDM3B* | *PCYT2* | *SLC38A3* | *WLS* |
| *BBS10* | *EIF4A3* | *KDM4B* | *PDE2A* | *SLC39A8* | *WNK3* |
| *BBS12* | *EIF5A* | *KDM5A* | *PDE4D* | *SLC44A1* | *WNT1* |
| *BBS2* | *ELAC2* | *KDM5B* | *PDE6D* | *SLC45A1* | *WWOX* |
| *BBS4* | *ELOVL4* | *KDM5C* | *PDGFRB* | *SLC46A1* | *XPA* |
| *BBS5* | *ELP2* | *KDM6A* | *PDHA1* | *SLC4A10* | *XRCC4* |
| *BBS7* | *EMC1* | *KDM6B* | *PDHB* | *SLC4A4* | *XYLT1* |
| *BBS9* | *EMC10* | *KIAA0586* | *PDHX* | *SLC5A7* | *YAP1* |
| *BCAP31* | *EMG1* | *KIAA0753* | *PDSS2* | *SLC6A1* | *YARS1* |
| *BCAS3* | *EML1* | *KIDINS220* | *PDZD8* | *SLC6A17* | *YIF1B* |
| *BCKDHA* | *EMX2* | *KIF11* | *PEPD* | *SLC6A19* | *YIPF5* |
| *BCKDHB* | *ENTPD1* | *KIF14* | *PET100* | *SLC6A3* | *YWHAE* |
| *BCKDK* | *EP300* | *KIF1A* | *PEX1* | *SLC6A8* | *YWHAG* |
| *BCL11A* | *EPG5* | *KIF21B* | *PEX10* | *SLC6A9* | *YY1* |
| *BCL11B* | *EPRS1* | *KIF2A* | *PEX11B* | *SLC9A6* | *ZBTB11* |
| *BCOR* | *ERBB4* | *KIF4A* | *PEX12* | *SLC9A7* | *ZBTB18* |
| *BCORL1* | *ERCC1* | *KIF5A* | *PEX13* | *SLF2* | *ZBTB20* |
| *BCS1L* | *ERCC2* | *KIF7* | *PEX16* | *SLITRK2* | *ZBTB21* |
| *BICRA* | *ERCC3* | *KIFBP* | *PEX2* | *SMAD4* | *ZBTB24* |
| *BLM* | *ERCC5* | *KLF7* | *PEX26* | *SMAD6* | *ZBTB7A* |
| *BLOC1S1* | *ERCC6* | *KLHL15* | *PEX5* | *SMARCA2* | *ZC4H2* |
| *BLTP1* | *ERCC6L2* | *KLHL20* | *PEX6* | *SMARCA4* | *ZDHHC9* |
| *BOD1* | *ERCC8* | *KLHL7* | *PEX7* | *SMARCA5* | *ZEB2* |
| *BOLA3* | *ERF* | *KMT2A* | *PGAP1* | *SMARCB1* | *ZFHX3* |
| *BPTF* | *ERI1* | *KMT2B* | *PGAP2* | *SMARCC2* | *ZFHX4* |
| *BRAF* | *ERLIN2* | *KMT2D* | *PGAP3* | *SMARCD1* | *ZFYVE26* |
| *BRAT1* | *ESAM* | *KMT2E* | *PGK1* | *SMARCE1* | *ZIC1* |
| *BRD4* | *ESCO2* | *KMT5B* | *PGM2L1* | *SMC1A* | *ZIC2* |
| *BRF1* | *ETFA* | *KPNA7* | *PGM3* | *SMC3* | *ZMIZ1* |
| *BRPF1* | *ETFB* | *KPTN* | *PHF21A* | *SMC5* | *ZMYM2* |
| *BRSK2* | *ETFDH* | *KRAS* | *PHF5A* | *SMG8* | *ZMYM3* |
| *BRWD3* | *ETHE1* | *L1CAM* | *PHF6* | *SMG9* | *ZMYND11* |
| *BSCL2* | *EXOC7* | *L2HGDH* | *PHF8* | *SMOC1* | *ZMYND8* |
| *BUB1B* | *EXOC8* | *LAGE3* | *PHGDH* | *SMPD1* | *ZNF142* |
| *BUD13* | *EXOSC2* | *LAMA1* | *PHIP* | *SMPD4* | *ZNF148* |
| *C12orf4* | *EXOSC3* | *LAMA2* | *PI4KA* | *SMS* | *ZNF292* |
| *C12orf57* | *EXOSC5* | *LAMB1* | *PIBF1* | *SNAP25* | *ZNF335* |
| *C2orf69* | *EXOSC8* | *LAMC3* | *PIDD1* | *SNAP29* | *ZNF407* |
| *CA2* | *EXOSC9* | *LAMP2* | *PIEZO2* | *SNAPC4* | *ZNF462* |
| *CA5A* | *EXT2* | *LARGE1* | *PIGA* | *SNIP1* | *ZNF526* |
| *CA8* | *EXTL3* | *LARP7* | *PIGB* | *SNRPB* | *ZNF699* |
| *CACNA1A* | *EZH1* | *LARS1* | *PIGC* | *SNX14* | *ZNF711* |
| *CACNA1B* | *EZH2* | *LARS2* | *PIGG* | *SNX27* | *ZNHIT3* |
| *CACNA1C* | *FAM111A* | *LAS1L* | *PIGH* | *SON* | *ZSWIM6* |
| *CACNA1D* | *FAM126A* | *LEO1* | *PIGK* | *SOS1* |  |
| *CACNA1E* | *FAM149B1* | *LETM1* | *PIGL* | *SOS2* |  |
| *CACNA1G* | *FAM50A* | *LHX2* | *PIGN* | *SOX10* |  |
| *CACNA1H* | *FANCD2* | *LIAS* | *PIGO* | *SOX11* |  |
| *CACNA1I* | *FAR1* | *LIG4* | *PIGP* | *SOX2* |  |
| *CACNA2D1* | *FARS2* | *LINGO1* | *PIGQ* | *SOX3* |  |
| *CACNA2D2* | *FARSA* | *LINS1* | *PIGS* | *SOX4* |  |
| *CAD* | *FASTKD2* | *LIPT1* | *PIGT* | *SOX5* |  |
| *CAMK2A* | *FAT1* | *LIPT2* | *PIGU* | *SOX6* |  |
| *CAMK2B* | *FAT4* | *LMBRD1* | *PIGV* | *SPART* |  |
| *CAMK4* | *FBN3* | *LMBRD2* | *PIGW* | *SPATA5* |  |
| *CAMSAP1* | *FBRSL1* | *LMNB1* | *PIGY* | *SPATA5L1* |  |
| *CAMTA1* | *FBXL3* | *LMNB2* | *PIK3CA* | *SPECC1L* |  |
| *CAMTA2* | *FBXL4* | *LNPK* | *PIK3R1* | *SPEN* |  |
| *CAPN10* | *FBXO11* | *LONP1* | *PIK3R2* | *SPG11* |  |
| *CAPN15* | *FBXO28* | *LRP2* | *PIP5K1C* | *SPOP* |  |
| *CAPRIN1* | *FBXO31* | *LRPPRC* | *PISD* | *SPR* |  |
| *CAPZA2* | *FBXW11* | *LSM1* | *PITRM1* | *SPRED1* |  |
| *CARS1* | *FBXW7* | *LSS* | *PLA2G6* | *SPRED2* |  |
| *CARS2* | *FCSK* | *LTBP1* | *PLAA* | *SPTAN1* |  |
| *CASK* | *FDFT1* | *LYRM7* | *PLCB1* | *SPTBN1* |  |
| *CBL* | *FGD1* | *LZTFL1* | *PLCH1* | *SPTBN2* |  |
| *CBS* | *FGF12* | *LZTR1* | *PLK4* | *SPTBN4* |  |
| *CBX1* | *FGF13* | *MAB21L1* | *PLP1* | *SQSTM1* |  |
| *CBY1* | *FGFR1* | *MAB21L2* | *PLPBP* | *SRCAP* |  |
| *CC2D1A* | *FGFR2* | *MACF1* | *PLXNA1* | *SRD5A3* |  |
| *CC2D2A* | *FGFR3* | *MADD* | *PLXNA3* | *SRP54* |  |
| *CCBE1* | *FH* | *MAF* | *PMM2* | *SRRM2* |  |
| *CCDC115* | *FIBP* | *MAG* | *PMPCA* | *SRSF1* |  |
| *CCDC22* | *FIG4* | *MAGEL2* | *PMPCB* | *ST3GAL3* |  |
| *CCDC32* | *FILIP1* | *MAN1B1* | *PNKP* | *ST3GAL5* |  |
| *CCDC47* | *FITM2* | *MAN2B1* | *PNPLA6* | *STAG1* |  |
| *CCDC82* | *FKRP* | *MAN2C1* | *PNPO* | *STAG2* |  |
| *CCDC88A* | *FKTN* | *MANBA* | *PNPT1* | *STAMBP* |  |
| *CCDC88C* | *FLNA* | *MAOA* | *POGZ* | *STEEP1* |  |
| *CCND2* | *FMN2* | *MAP1B* | *POLA1* | *STIL* |  |
| *CDC42* | *FMR1* | *MAP2K1* | *POLG* | *STIM1* |  |
| *CDC42BPB* | *FOLR1* | *MAP2K2* | *POLR1A* | *STRA6* |  |
| *CDH11* | *FOSL2* | *MAP3K7* | *POLR1C* | *STRADA* |  |
| *CDH2* | *FOXG1* | *MAP4K4* | *POLR2A* | *STT3A* |  |
| *CDK10* | *FOXP1* | *MAPK1* | *POLR3A* | *STUB1* |  |
| *CDK13* | *FOXP2* | *MAPK8IP3* | *POLR3B* | *STX1B* |  |
| *CDK16* | *FOXP4* | *MAPKAPK5* | *POLR3K* | *STXBP1* |  |
| *CDK19* | *FOXRED1* | *MASP1* | *POLRMT* | *SUCLA2* |  |
| *CDK5RAP2* | *FRA10AC1* | *MAST1* | *POMGNT1* | *SUFU* |  |
| *CDK6* | *FRAS1* | *MAST3* | *POMGNT2* | *SUOX* |  |
| *CDK8* | *FRMD4A* | *MAST4* | *POMK* | *SUPT16H* |  |
| *CDK9* | *FRMD5* | *MAT1A* | *POMT1* | *SUPV3L1* |  |
| *CDKL5* | *FRMPD4* | *MBD5* | *POMT2* | *SURF1* |  |
| *CDON* | *FRRS1L* | *MBOAT7* | *POR* | *SUZ12* |  |
| *CELF2* | *FRY* | *MBTPS2* | *PORCN* | *SVBP* |  |
| *CENPE* | *FTO* | *MCCC2* | *POU3F2* | *SYN1* |  |
| *CENPF* | *FTSJ1* | *MCM3AP* | *POU3F3* | *SYNCRIP* |  |
| *CENPJ* | *FUCA1* | *MCM6* | *POU4F1* | *SYNE1* |  |
| *CEP104* | *FUT8* | *MCOLN1* | *PPFIA3* | *SYNGAP1* |  |
| *CEP120* | *FZR1* | *MCPH1* | *PPFIBP1* | *SYNJ1* |  |
| *CEP135* | *GABBR1* | *MDH2* | *PPIL1* | *SYP* |  |
| *CEP152* | *GABBR2* | *MECP2* | *PPM1D* | *SYT1* |  |
| *CEP19* | *GABRA1* | *MED11* | *PPOX* | *SZT2* |  |
| *CEP290* | *GABRA2* | *MED12* | *PPP1CB* | *TAB2* |  |
| *CEP41* | *GABRA3* | *MED12L* | *PPP1R12A* | *TACO1* |  |
| *CEP55* | *GABRA5* | *MED13* | *PPP1R15B* | *TAF1* |  |
| *CEP57* | *GABRB2* | *MED13L* | *PPP1R21* | *TAF13* |  |
| *CEP63* | *GABRB3* | *MED17* | *PPP2CA* | *TAF2* |  |
| *CEP83* | *GABRD* | *MED23* | *PPP2R1A* | *TAF4* |  |
| *CEP85L* | *GABRG2* | *MED25* | *PPP2R5D* | *TAF6* |  |
| *CERT1* | *GAD1* | *MED27* | *PPP3CA* | *TAF8* |  |
| *CHAMP1* | *GALC* | *MEF2C* | *PQBP1* | *TAFAZZIN* |  |
| *CHD1* | *GALNT2* | *MEGF8* | *PRDM13* | *TANC2* |  |
| *CHD2* | *GALT* | *MEIS2* | *PRDM15* | *TANGO2* |  |
| *CHD3* | *GAMT* | *MESD* | *PREPL* | *TAOK1* |  |
| *CHD4* | *GAN* | *METTL23* | *PRICKLE2* | *TARS2* |  |
| *CHD5* | *GATA6* | *METTL5* | *PRKACB* | *TASP1* |  |
| *CHD7* | *GATAD2A* | *MFF* | *PRKAR1B* | *TAT* |  |
| *CHD8* | *GATAD2B* | *MFSD2A* | *PRKCG* | *TBC1D20* |  |
| *CHKA* | *GATM* | *MGAT2* | *PRKRA* | *TBC1D23* |  |
| *CHKB* | *GBA2* | *MGME1* | *PRMT7* | *TBC1D24* |  |
| *CHMP1A* | *GCDH* | *MICOS13* | *PRODH* | *TBC1D2B* |  |
| *CIC* | *GCH1* | *MICU1* | *PRORP* | *TBC1D7* |  |
| *CIT* | *GCSH* | *MID1* | *PROSER1* | *TBCD* |  |
| *CKAP2L* | *GDF1* | *MINPP1* | *PRPF8* | *TBCE* |  |
| *CLCN2* | *GDF11* | *MKKS* | *PRPS1* | *TBCEL* |  |
| *CLCN3* | *GDI1* | *MLC1* | *PRR12* | *TBCK* |  |
| *CLCN4* | *GEMIN4* | *MLYCD* | *PRR14L* | *TBL1XR1* |  |
| *CLCN6* | *GEMIN5* | *MMAA* | *PRRT2* | *TBR1* |  |
| *CLCN7* | *GFAP* | *MMACHC* | *PRUNE1* | *TBX2* |  |
| *CLDN11* | *GFM1* | *MMADHC* | *PSMC3* | *TCEAL1* |  |
| *CLDN5* | *GFM2* | *MMUT* | *PSMD11* | *TCF12* |  |
| *CLP1* | *GJC2* | *MN1* | *PSMD12* | *TCF20* |  |
| *CLPB* | *GK* | *MOCS1* | *PSMD6* | *TCF4* |  |
| *CLPP* | *GLB1* | *MOCS2* | *PSPH* | *TCF7L2* |  |
| *CLTC* | *GLDC* | *MOGS* | *PTCH1* | *TCTN2* |  |
| *CNKSR1* | *GLI2* | *MORC2* | *PTCHD1* | *TDP2* |  |
| *CNKSR2* | *GLI3* | *MPC1* | *PTDSS1* | *TECPR2* |  |
| *CNNM2* | *GLIS3* | *MPC2* | *PTEN* | *TECR* |  |
| *CNOT1* | *GLRA2* | *MPDU1* | *PTPA* | *TEFM* |  |
| *CNOT3* | *GLS* | *MPDZ* | *PTPN11* | *TELO2* |  |
| *CNOT9* | *GLUL* | *MPLKIP* | *PTPN23* | *TENM3* |  |

**Supplementary Table 2.** Clinical and molecular characteristics associated with VUS in patients with TS. Abbreviations: MAF, minor allele frequency; CADD score: Combined Annotation Dependent Depletion prediction score; N/A, not available due to absence of the variant in population databases.

| Patient ID | Gene(s) involved | Type of variant(s) | MAF | CADD score | Clinical features |
| --- | --- | --- | --- | --- | --- |
| #12 | *TREX1* | Heterozygous frameshift deletion, c.409del p.(Glu137SerfsTer23) in *TREX1* gene | N/A | N/A | Motor and vocal tics |
| #13 | *PRKRA* | Heterozygous missense SNV, c.665C>T p.(Pro222Leu) in *PRKRA* gene | 0.0001 | 21.8 | Motor and vocal tics |
| #14 | *GRIA3* | Heterozygous missense SNV, c.1259C>T p.(Ser420Leu) in *GRIA3* gene | N/A | 6 | Motor tics |
| #15 | *PLXNA3* | Heterozygous missense SNV, c.1114G>C p.(Glu372Gln) in PLXNA3 gene | 0.00001 | 18 | Motor tics, anxiety |
| #16 | *SHANK2* | Heterozygous missense SNV, c.4900G>A p.(Gly1634Ser) in *SHANK2* gene | N/A | 23.3 | Motor and vocal tics, autism spectrum disorder, anxiety |
| #17 | ADNP; AFF2 (FMR2); GIGYF1 | Heterozygous missense SNV, c.2960A>G p.(Asn987Ser) in *ADNP* gene; Heterozygous missense SNV, c.3589G>A p.(Ala1197Thr) in *AFF2* gene; Heterozygous missense SNV, c.2278C>T p.(Leu760Phe) in *GIGYF1* gene | 0.000020; 0.000020; 0.000002 | 18.31; 1.70; 20.5 | Motor tics |
| #18 | *ANKRD17; DPYD* | Heterozygous missense SNV, c.3185A>G p.(Asn1062Ser) in *ANKRD17* gene; Heterozygous splice-site SNV, c.1905+1G>A in *DPYD* gene | N/A; 0.00569 | 25.0; 32.0 | Motor and vocal tics |
| #19 | *DDX3X* | Hemizygous missense SNV, c.293G>C p.(Ser98Thr) in *DDX3X* gene | N/A | 19.85 | Motor tics, learning difficulties |
| #20 | *SPTBN1* | Heterozygous missense SNV, c.5726A>T p.(Asp1909Val) in *SPTBN1* gene | N/A | 26.3 | Motor tics |
| #21 | *SETD2* | Heterozygous missense SNV, c.3809C>T p.(Thr1270Met) in *SETD2* gene | 0.00001 | 26.5 | Motor tics, dysmorphic features |
| #22 | *RERE; MMACHC; PNPT1; CENPF* | Heterozygous missense SNV, c.1763G>A p.(Arg588Gln) in *RERE* gene; Heterozygous frameshift duplication, c.271dup p.(Arg91LysfsTer14) in *MMACHC* gene; Heterozygous splice-site SNV, c.2014-2A>G in *PNPT1* gene; Heterozygous splice-site SNV, c.8962+2T>C in *CENPF* gene | 0.00001; 0.00112; 0.000004; 0.00001 | 32; N/A; 22; 18 | Motor tics, anxiety |
| #23 | CAMK4; COG5 | Heterozygous stop-gain SNV, c.1414G>T p.(Glu472Ter) in *CAMK4* gene; Heterozygous frameshift deletion, c.704_706delinsTAGTGGAATT p.(Ala235ValfsTer6) in *COG5* gene | 0.00005; 0.00001 | 39.0; N/A | Motor tics |
| #24 | *MED13; DCHS1; KIAA0753; SLC25A1; MTO1* | Heterozygous missense SNV, c.599C>G p.(Ser200Cys) in *MED13* gene; Heterozygous missense SNV, c.6988C>T p.(Arg2330Cys) in *DCHS1* gene; Heterozygous splice-site SNV, c.1315+1G>T in *KIAA0753* gene; Heterozygous missense SNV, c.740G>A p.(Arg247Gln) in *SLC25A1* gene; Heterozygous nonsense SNV, c.1996C>T p.(Arg666Ter) in *MTO1* gene | N/A; 0.00002; 0.00001; 0.00002; 0.00010 | 28.1; 27.0; 33.0; 25.1; 36.0 | Motor and vocal tics |
| #25 | *KIF11, GATM, ALG6* | Heterozygous missense SNV, c.2666A>T p.(Asp889Val) in *KIF11* gene Heterozygous missense SNV, c.1237C>T p.(Arg413Trp) in *GATM* gene; Heterozygous missense SNV, c.482A>G p.(Tyr161Cys) in *ALG6* gene | N/A; N/A; 0.00008 | 14; 28; 20 | Motor and vocal tics, congenital strabismus |
| #26 | *SOS1; SOS2; SETBP1; SPG11* | Heterozygous missense SNV, c.755T>C p.(Ile252Thr) in *SOS1* gene; Heterozygous frameshift deletion, c.3125_3126del p.(Thr1042ArgfsTer23) in *SOS2* gene; Heterozygous missense SNV, c.3349C>A p.(Leu1117Met) in *SETBP1* gene; Heterozygous frameshift duplication, c.5986dup p.(Cys1996LeufsTer4) in *SPG11* gene | 0.00008; N/A; N/A; 0.00002 | 25.7; N/A; 24.4; N/A | Motor and vocal tics, autism spectrum disorder |
| #27 | *ASH1L, EIF3F, MUT* | Heterozygous missense SNV, c.2576C>A p.(Ser859Tyr) in *ASH1L* gene; Heterozygous missense SNV, c.694T>G p.(Phe232Val) in *EIF3F* gene; Heterozygous missense SNV, c.643G>A p.(Gly215Ser) in *MUT* gene | N/A; 0.00071; N/A | 27.2; 28.5; 28.7 | Motor and vocal tics, autism spectrum disorder, attention-Deficit/Hyperactivity Disorder |
| #28 | *NIPBL* | Heterozygous missense SNV, c.7166G>A p.(Ser2389Asn) in *NIPBL* gene | N/A | N/A | Motor tics, anxiety |
| #29 | POU3F3; NEB | Heterozygous missense SNV, c.1091A>G p.(Glu364Gly) in *POU3F3* gene; Heterozygous splice-site SNV, c.11910+2T>G in *NEB* gene | N/A | 29.8; 34.0 | Motor tics, attention-deficit/hyperactivity disorder |
| #30 | *RELN* | Heterozygous missense SNV, c.7561G>A p.(Ala1921Thr) in *RELN* gene | N/A | N/A | Motor tics, learning difficulties |
| #31 | *RELN* | Heterozygous frameshift deletion, c.4899_4902del p.(Ser1634TrpfsTer5) in *RELN* gene | N/A | N/A | Motor tics, obsessive–compulsive disorder |
| #32 | *CHRNA4* | Heterozygous missense SNV, c.890C>T p.(Pro297Leu) in *CHRNA4* gene | 0.000004 | 31 | Motor tics, anxiety |
| #33 | ZMYM2; PNKD | Heterozygous nonsense *de novo* SNV, c.1039C>T p.(Arg347Ter) in *ZMYM2* gene; Heterozygous nonsense indel, c.1107_1108delCCinsGT p.(Gln370Ter) in *PNKD* gene | N/A | N/A | Motor tics |
| #34 | *PLXNA3* | Hemizygous missense SNV, c.853C>T p.(Arg285Cys) in *PLXNA3* gene | N/A | N/A | Motor tics, anxiety |
| #35 | *TNRC6B* | Heterozygous missense *de novo* SNV, c.1772G>A p.(Arg591Gln) in *TNRC6B* gene | N/A | N/A | Motor tics, anxiety |
| #36 | *PHIP* | Heterozygous *de novo* multi-exonic deletion in *PHIP gene* | N/A | N/A | Motor tics |
| #37 | *CUX2, KIF21B, AGMO, SRCAP* | Heterozygous missense SNVs (in *CUX2 and SRCAP genes*); heterozygous splice-site variants (in *KIF21B and* *AGMO genes*) | N/A | N/A | Motor tics |
| #38 | *CSNK2B, RELN* | Heterozygous missense SNV, c.461C>T p.(Thr154Met) in *CSNK2B* gene; Heterozygous missense SNV, c.5764A>G p.(Thr1922Ala) in *RELN* gene | 0.000004; 0.00001 | 25.9; 25.7 | Motor tics, obsessive–compulsive disorder |
| #39 | *CACNA1A* | Heterozygous *de novo* missense SNP, c.3158A>G p.(Asp1053Gly) in *CACNA1A* gene | N/A | N/A | Motor tics, obsessive–compulsive disorder |
| #40 | *CWF19L1* | Heterozygous nonsense SNV, c.349G>T p.(Glu117Ter) in *CWF19L1* gene | N/A | N/A | Motor tics |

**Supplementary Table 3.** Clinical and molecular characteristics associated with NC-Vs in patients with TS. Abbreviations: MAF, minor allele frequency; CADD score: Combined Annotation Dependent Depletion prediction score; N/A, not available due to absence of the variant in population databases.

| Patient ID | Gene(s) involved | Type of variant(s) | MAF | CADD score | Clinical features |
| --- | --- | --- | --- | --- | --- |
| #41 | *CBS; KIAA0586; RARS2* | Heterozygous missense, c.833T>C p.(Ile278Thr) in *CBS* gene;Heterozygous frameshift deletion, c.428del p.(Arg143LysfsTer4) in *KIAA0586* gene and c.1629_1630del p.(Asp543GlufsTer4) in *RARS2* gene | 0.00083; N/A; 0.00312 | 22.2; N/A; N/A | Motor tics |
| #42 | *ALG1* | Heterozygous splice-site variant, c.1187+1G>A in *ALG1* gene | 0.00002 | 19.14 | Motor tics, attention-deficit/hyperactivity disorder |
| #43 | *ERI1; MMACHC* | Heterozygous splice-site variant, c.109-2A>G in *ERI1* gene; Heterozygous frameshift duplication, c.271dup p.(Arg91LysfsTer14) in *MMACHC* gene | 0.00028; 0.00112 | 32.0; N/A | Motor tics, obsessive–compulsive disorder, autism spectrum disorder |
| #44 | *MCCC2; VPS13A* | Heterozygous missense SNV, c.1015G>A p.(Val339Met) in *MCCC2* gene; Heterozygous frameshift deletion, c.6309_6313del p.(Ile2104AlafsTer24) in *VPS13A* gene | 0.00074; N/A | 32.0; N/A | Motor tics, obsessive–compulsive disorder |
| #45 | *CACNA1H; DBT* | Heterozygous nonsense variant, c.6421C>T p.(Gln2141Ter) in *CACNA1H* gene; Heterozygous nonsense variant, c.505C>T p.(Arg169Ter) in *DBT* gene | N/A | 45.0; 37.0 | Motor tics, epilepsy, autism spectrum disorder, behavioral disorder |
| #46 | *HEATR3; DBT* | Heterozygous splice-site SNV, c.1599+1G>C in *HEATR3* gene; Heterozygous nonsense SNV, c.505C>T p.(Arg169Ter) in *DBT* gene | N/A | 34.0; 37.0 | Motor tics, autism spectrum disorder, Arnold–Chiari type I malformation, obesity, behavioral disorder |
| #47 | *ADD1; ACADM* | Heterozygous missense c.731C>G p.(Ala244Gly) in *ADD1* gene and c.985A>G p.(Lys329Glu) in *ACADM* gene | N/A; 0.00333 | 28.2; 22.9 | Motor tics |
| #48 | *ATP7B* | Heterozygous splice-site SNV, c.1285+5G>T in *ATP7B* gene | 0.00006 | 19 | Motor tics |
| #49 | *AGMO* | Heterozygous splice-site SNV, c.677-1G>C in *AGMO* gene | 0.00001 | 34 | Motor tics, obsessive–compulsive disorder, attention-deficit/hyperactivity disorder |
| #50 | *HLCS; TASP1* | Heterozygous frameshift deletion/insertion, c.128_144delinsTTGCTTGAGATTAAGCCTGAGATTAAGG p.(Pro43LeufsTer3) in *HLCS* gene; Heterozygous frameshift deletion, c.1072del p.(Ser358ProfsTer8) in *TASP1* gene | N/A; N/A | N/A; N/A | Motor tics, obsessive–compulsive disorder |
| #51 | *DHCR7; DHPS; FMN2* | Heterozygous nonsense SNV, c.452G>A p.(Trp151Ter) in *DHCR7* gene; Heterozygous splice-site SNV, c.1014+1G>A in *DHPS* gene; Heterozygous frameshift deletion, c.5016del p.(Phe1672LeufsTer17) in *FMN2* gene | 0.00078; 0.00043; N/A | 37.0; 33.0; N/A | Motor tics |
| #52 | *ADCK3 (COQ8A); ALMS1* | Heterozygous missense SNV, c.1000C>T p.(Arg334Trp) in *ADCK3* (*COQ8A*) gene; Heterozygous frameshift duplication, c.7766dup p.(Thr2590AsnfsTer3) in *ALMS1* gene | 0.00006; N/A | 26; N/A | Motor tics, anxiety |
| #53 | *DPYD* | Heterozygous frameshift deletion, c.1109_1110del p.(Ile370LysfsTer5) in *DPYD* gene | 0.000004 | N/A | Motor tics, attention-deficit/hyperactivity disorder, autism spectrum disorder |
| #54 | *IARS (IARS1); TTI2* | Heterozygous frameshift deletion, c.1633_1634del p.(Lys545GlufsTer4) in *IARS* gene; Heterozygous splice-site variant, c.646_647+2del in *TTI2* gene | 0.000004; 0.00001 | N/A | Motor tics, obsessive–compulsive disorder, anxiety, enuresis |
| #55 | *TMEM216* | Heterozygous missense SNV, c.218G>T p.(Arg73Leu) in *TMEM216* gene | 0.00016 | 24 | Motor tics, behavioral disorder |
| #56 | *DPYD* | Heterozygous splice-site SNV, c.1905+1G>A in *DPYD* gene | 0.00569 | 33 | Motor tics |
| #57 | ALG1; GEMIN5 | Heterozygous nonsense SNV, c.543C>G p.(Tyr181Ter) in *ALG1* gene; Heterozygous splice-site SNV, c.328-2A>C in *GEMIN5* gene | N/A | 36.0; 33.0 | Motor tics, obsessive–compulsive disorder |
| #58 | *RTN4IP1; NDUFV1* | Heterozygous splice-site SNV, c.806+1G>A in *RTN4IP1* gene; Heterozygous splice-site SNV, c.156-2A>G in *NDUFV1* gene | 0.00002; 0.00001 | 34.0; 34.0 | Motor tics, obsessive–compulsive disorder, anxiety, headache, enthesitis/arthritis |
| #59 | *CHD8; RTN4IP1* | Heterozygous missense SNV, c.6922G>C p.(Asp2308His) in *CHD8* gene; Heterozygous splice-site SNV, c.806+1G>A in *RTN4IP1* gene | N/A; 0.00002 | 23.9; 34.0 | Motor tics, obsessive–compulsive disorder, anxiety, behavioral disorder, specific learning disorder |
| #60 | *NDUFV1* | Heterozygous splice-site SNV, c.156-2A>G in *NDUFV1* gene | 0.00001 | 34 | Motor tics, obsessive–compulsive disorder, specific learning disorder, nutcracker syndrome |
| #61 | *PAH* | Heterozygous nonsense SNV, c.561G>A p.(Trp187Ter) in *PAH* gene | 0.00001 | 32 | Motor tics, attention-deficit/hyperactivity disorder, specific learning disorder |
| #62 | *CAMTA1; BBS12; POR* | Heterozygous nonsense SNV, c.2944C>T p.(Arg982Ter) in *CAMTA1* gene; Heterozygous frameshift duplication, c.362dup p.(Asn121LysfsTer4) in *BBS12* gene; Heterozygous splice-site SNV, c.188+2T>C in *POR* gene | N/A; N/A; 0.000004 | 40.0; N/A; 24.4 | Motor tics, obsessive–compulsive disorder, anxiety |
| #63 | *RERE; NBEA; B3GALTL (B3GLCT)* | Heterozygous missense SNV, c.3325C>A p.(Pro1109Thr) in RERE gene; Heterozygous missense SNV, c.5126C>G p.(Ser1709Cys) in NBEA gene; Heterozygous nonsense SNV, c.1098T>A p.(Tyr366Ter) in B3GALTL gene | N/A | 24.0; 22.3; 34.0 | Motor tics, behavioral disorder |
| #64 | *CEP152* | Heterozygous nonsense SNV, c.2920C>T p.(Gln974Ter) in *CEP152* gene | 0.0000007 | 39 | Motor tics |
| #65 | *GDF11; PAH* | Heterozygous frameshift deletion, c.136del p.(Glu46SerfsTer31) in *GDF11* gene; Heterozygous missense SNV, c.1208C>T p.(Ala403Val) in *PAH* gene | 0.00000; 0.00058 | N/A; 26.3 | Motor tics, behavioral disorder |
| #66 | *PEX16* | Heterozygous frameshift deletion, c.644del p.(Leu215ArgfsTer40) in *PEX16* gene | N/A | N/A | Motor tics, language disorder |
| #67 | *AGPS; CEP290* | Heterozygous splice-site SNV, c.1856-1G>A in *AGPS* gene; Heterozygous frameshift deletion, c.5493del p.(Ala1832ProfsTer19) in *CEP290* gene | N/A; 0.00002 | 34.0; N/A | Motor tics, obsessive–compulsive disorder, developmental coordination disorder |
| #68 | *PAH; POR* | Heterozygous frameshift deletion, c.165del p.(Phe55LeufsTer6) in *PAH* gene; Heterozygous frameshift duplication, c.1685_1688dup p.(Leu564AspfsTer12) in *POR* gene | 0.00001; 0.000004 | N/A | Motor tics, attention-deficit/hyperactivity disorder |
| #69 | *ITSN1; DIS3L2* | Heterozygous missense SNV, c.730C>A p.(Gln244Lys) in *ITSN1* gene; Heterozygous frameshift duplication, c.582dup p.(Val195SerfsTer5) in *DIS3L2* gene | N/A | 24; N/A | Motor tics, obsessive–compulsive disorder, behavioral disorder, pituitary dwarfism, selective mutism, psychotic features |
| #70 | ITM2B; MBD5 | Heterozygous missense SNV, c.728G>A p.(Arg243His) in *ITM2B* gene; Heterozygous missense SNV, c.4208T>C p.(Leu1403Ser) in *MBD5* gene | 0.00003; N/A | 25.9; 27.5 | Motor tics, severe behavioral disorder, autism spectrum disorder |
| #71 | *WWOX* | Heterozygous frameshift deletion, c.1043del p.(Phe348SerfsTer57) in *WWOX* gene | 0.00001 | N/A | Motor tics |
| #72 | *BBS1* | Heterozygous missense SNV, c.1169T>G p.(Met390Arg) in *BBS1* gene | 0.00157 | 19 | Motor tics, obsessive–compulsive disorder, specific learning disorder |
| #73 | *EIF2B2, NEMF, RIMS1* | Heterozygous frameshift deletion in *EIF2B2*; heterozygous nonsense SNV in *NEMF*; heterozygous missense SNV in *RIMS1* | N/A; 0.00004; N/A | N/A; 37.0; N/A | Motor tics, obsessive–compulsive disorder, anxiety, headache |
| #74 | *PNPT1* | Heterozygous missense SNP, c.1519G>T p.(Ala507Ser) in *PNPT1* gene | 0.00021 | 25 | Motor tics, obsessive–compulsive disorder, anxiety, microcephaly |
| #75 | *AGTPBP1, CLN5, MMACHC, PAH* | Heterozygous frameshift indel, c.2494_2497delinsTTCAAA p.(Met832PhefsTer13) in *AGTPBP1* gene; Heterozygous frameshift indel, c.271dup p.(Arg91LysfsTer14) in *MMACHC* gene; Heterozygous nonsense indel, c.672del p.(Trp224Ter) in *CLN5* gene; Heterozygous missense SNP, c.1208C>T p.(Ala403Val) in *PAH* gene | N/A | N/A | Motor tics, obsessive–compulsive disorder, enuresis, specific learning disorder |
| #76 | *GALT* | Heterozygous missense SNP, c.292G>A p.(Asp98Asn) in *GALT* gene | 0.00004 | 32 | Motor tics |
| #77 | *TRIO* | Heterozygous missense SNP, c.3372G>T p.(Leu1124Phe) in *TRIO* gene | 0.000004 | 22.6 | Motor tics, autism spectrum disorder |
| #78 | *MYT1L, KIAA1033, PCNT* | Heterozygous copy-number duplication, exons 6–25 in *MYT1L* gene; Heterozygous nonsense SNV, c.2680C>T p.(Arg894Ter) in *KIAA1033* gene; Heterozygous frameshift deletion, c.4224del p.(Lys1409ArgfsTer6) in *PCNT* gene; Heterozygous splice-site SNV, c.3608-2A>G in *PCNT* gene | N/A; 0.00001; N/A; N/A | N/A; 47.0; N/A; 25.4 | Motor tics, anxiety, autism spectrum disorder, behavioral disorder |
| #79 | *MVK* | Heterozygous missense SNV, c.1129G>A p.(Val377Ile) in *MVK* gene | 0.00158 | 13.96 | Motor tics, obsessive–compulsive disorder |
| #80 | *ASL, BBS5, PCNT* | Heterozygous missense SNV, c.707G>A p.(Arg236Gln) in *ASL* gene; Heterozygous splicing SNV, c.681+1G>T in *BBS5* gene; Heterozygous frameshift indel, c.9752del p.(Pro3251GlnfsTer39) in *PCNT* gene | 0.00001; N/A; N/A | 28.7; 35.0; N/A | Motor tics, attention-deficit/hyperactivity disorder |

**Supplementary Table 4.** Genetic mapping statistics per individual exome. For each proband, the table summarizes the number and type of variants, including the total number of reported variants, number of single-nucleotide variants (SNVs) and insertions/deletions (indels), number of rare variants (MAF < 1%), and number of coding variants.

| ID | N. Variants | N. of SNPs | N. of indels | N. rare variants | N. coding variants |
| --- | --- | --- | --- | --- | --- |
| #1 | 3 | 2 | 1 | 3 | 2 |
| #2 | 3 | 1 | 2 | 3 | 3 |
| #3 | 1 | 1 | 0 | 1 | 1 |
| #4 | 1 | 1 | 0 | 1 | 1 |
| #5 | 1 | 1 | 0 | 1 | 1 |
| #6 | 2 | 1 | 1 | 2 | 2 |
| #7 | 4 | 3 | 0 | 3 | 3 |
| #8 | 1 | 1 | 0 | 1 | 1 |
| #9 | 1 | 1 | 0 | 1 | 1 |
| #10 | 1 | 1 | 0 | 1 | 1 |
| #11 | 2 | 1 | 1 | 2 | 2 |
| #12 | 1 | 0 | 1 | 1 | 1 |
| #13 | 1 | 1 | 0 | 1 | 1 |
| #14 | 1 | 1 | 0 | 1 | 1 |
| #15 | 1 | 1 | 0 | 1 | 1 |
| #16 | 1 | 1 | 0 | 1 | 1 |
| #17 | 3 | 3 | 0 | 3 | 3 |
| #18 | 2 | 1 | 0 | 2 | 1 |
| #19 | 1 | 1 | 0 | 1 | 1 |
| #20 | 1 | 1 | 0 | 1 | 1 |
| #21 | 1 | 1 | 0 | 1 | 1 |
| #22 | 4 | 1 | 1 | 4 | 4 |
| #23 | 2 | 1 | 1 | 2 | 2 |
| #24 | 5 | 5 | 0 | 5 | 4 |
| #25 | 3 | 3 | 0 | 3 | 3 |
| #26 | 4 | 2 | 2 | 4 | 4 |
| #27 | 3 | 3 | 0 | 3 | 3 |
| #28 | 1 | 1 | 0 | 1 | 1 |
| #29 | 1 | 2 | 0 | 2 | 1 |
| #30 | 1 | 1 | 0 | 1 | 1 |
| #31 | 1 | 0 | 1 | 1 | 1 |
| #32 | 1 | 1 | 0 | 1 | 1 |
| #33 | 2 | 1 | 1 | 2 | 2 |
| #34 | 1 | 1 | 0 | 1 | 1 |
| #35 | 1 | 1 | 0 | 1 | 1 |
| #36 | 1 | 0 | 1 | 1 | 1 |
| #37 | 4 | 3 | 1 | 4 | 2 |
| #38 | 2 | 2 | 0 | 2 | 2 |
| #39 | 1 | 1 | 0 | 1 | 1 |
| #40 | 1 | 1 | 0 | 1 | 1 |
| #41 | 3 | 1 | 2 | 3 | 3 |
| #42 | 1 | 1 | 0 | 1 | 0 |
| #43 | 2 | 1 | 1 | 2 | 1 |
| #44 | 2 | 1 | 1 | 2 | 2 |
| #45 | 2 | 2 | 0 | 2 | 2 |
| #46 | 2 | 2 | 0 | 2 | 1 |
| #47 | 2 | 2 | 0 | 2 | 2 |
| #48 | 1 | 1 | 0 | 1 | 0 |
| #49 | 1 | 1 | 0 | 1 | 0 |
| #50 | 2 | 0 | 2 | 2 | 2 |
| #51 | 3 | 2 | 1 | 3 | 3 |
| #52 | 2 | 1 | 1 | 2 | 2 |
| #53 | 1 | 0 | 1 | 1 | 1 |
| #54 | 2 | 0 | 2 | 2 | 1 |
| #55 | 1 | 1 | 0 | 1 | 1 |
| #56 | 1 | 1 | 0 | 1 | 1 |
| #57 | 2 | 2 | 0 | 2 | 2 |
| #58 | 2 | 2 | 0 | 2 | 2 |
| #59 | 2 | 1 | 1 | 2 | 2 |
| #60 | 1 | 1 | 0 | 1 | 1 |
| #61 | 1 | 1 | 0 | 1 | 1 |
| #62 | 3 | 2 | 1 | 3 | 2 |
| #63 | 3 | 2 | 1 | 3 | 3 |
| #64 | 1 | 1 | 0 | 1 | 1 |
| #65 | 2 | 1 | 1 | 2 | 2 |
| #66 | 1 | 0 | 1 | 1 | 1 |
| #67 | 2 | 1 | 1 | 2 | 1 |
| #68 | 2 | 0 | 2 | 2 | 2 |
| #69 | 2 | 1 | 1 | 2 | 2 |
| #70 | 2 | 2 | 0 | 2 | 2 |
| #71 | 1 | 0 | 1 | 1 | 1 |
| #72 | 1 | 1 | 0 | 1 | 1 |
| #73 | 3 | 2 | 1 | 3 | 3 |
| #74 | 1 | 1 | 0 | 1 | 1 |
| #75 | 4 | 1 | 3 | 4 | 4 |
| #76 | 1 | 1 | 0 | 1 | 1 |
| #77 | 1 | 1 | 0 | 1 | 1 |
| #78 | 4 | 1 | 3 | 4 | 4 |
| #79 | 1 | 1 | 0 | 1 | 1 |
| #80 | 3 | 1 | 2 | 2 | 4 |
